# Serum anti-DIDO1, anti-CPSF2, and anti-FOXJ2 antibodies as predictive risk markers for acute ischemic stroke

**DOI:** 10.1186/s12916-021-02001-9

**Published:** 2021-06-09

**Authors:** Takaki Hiwasa, Hao Wang, Ken-ichiro Goto, Seiichiro Mine, Toshio Machida, Eiichi Kobayashi, Yoichi Yoshida, Akihiko Adachi, Tomoo Matsutani, Mizuki Sata, Kazumasa Yamagishi, Hiroyasu Iso, Norie Sawada, Shoichiro Tsugane, Mitoshi Kunimatsu, Ikuo Kamitsukasa, Masahiro Mori, Kazuo Sugimoto, Akiyuki Uzawa, Mayumi Muto, Satoshi Kuwabara, Yoshio Kobayashi, Mikiko Ohno, Eiichiro Nishi, Akiko Hattori, Masashi Yamamoto, Yoshiro Maezawa, Kazuki Kobayashi, Ryoichi Ishibashi, Minoru Takemoto, Koutaro Yokote, Hirotaka Takizawa, Takashi Kishimoto, Kazuyuki Matsushita, Sohei Kobayashi, Fumio Nomura, Takahiro Arasawa, Akiko Kagaya, Tetsuro Maruyama, Hisahiro Matsubara, Minako Tomiita, Shinsaku Hamanaka, Yushi Imai, Tomoo Nakagawa, Naoya Kato, Jiro Terada, Takuma Matsumura, Yusuke Katsumata, Akira Naito, Nobuhiro Tanabe, Seiichiro Sakao, Koichiro Tatsumi, Masaaki Ito, Fumiaki Shiratori, Makoto Sumazaki, Satoshi Yajima, Hideaki Shimada, Mikako Shirouzu, Shigeyuki Yokoyama, Takashi Kudo, Hirofumi Doi, Katsuro Iwase, Hiromi Ashino, Shu-Yang Li, Masaaki Kubota, Go Tomiyoshi, Natsuko Shinmen, Rika Nakamura, Hideyuki Kuroda, Yasuo Iwadate

**Affiliations:** 1 Department of Neurological Surgery, Graduate School of Medicine, Chiba University, Chiba, 260-8670 Japan; 2 Department of Biochemistry and Genetics, Graduate School of Medicine, Chiba University, Chiba, 260-8670 Japan; 3 Comprehensive Stroke Center, Chiba University Hospital, Chiba, 260-8677 Japan; 4 Department of Anesthesia, The First Affiliated Hospital, Jinan University, Guanzhou, 510632 P. R. China; 5 Department of Neurological Surgery, Chiba Prefectural Sawara Hospital, Chiba, 287-0003 Japan; 6 Department of Neurological Surgery, Chiba Cerebral and Cardiovascular Center, Chiba, 290-0512 Japan; 7 Department of Neurosurgery, Eastern Chiba Medical Center, Chiba, 283-8686 Japan; 8 Department of Public Health Medicine, Faculty of Medicine, and Health Services Research and Development Center, University of Tsukuba, Tsukuba, 305-8575 Japan; 9 Department of Preventive Medicine and Public Health, Keio University School of Medicine, Tokyo, 160-8582 Japan; 10 Public Health, Department of Social Medicine, Osaka University Graduate School of Medicine, Suita, 565-0871 Japan; 11 Epidemiology and Prevention Group, Center for Public Health Sciences, National Cancer Center, Tokyo, 104-0045 Japan; 12 Department of Home Economics, Nagoya Women’s University, Nagoya, 467-8610 Japan; 13 Department of Neurology, Chiba Rosai Hospital, Chiba, 290-0003 Japan; 14 Department of Neurology, Chibaken Saiseikai Narashino Hospital, Chiba, 275-8580 Japan; 15 Department of Neurology, Graduate School of Medicine, Chiba University, Chiba, 260-8670 Japan; 16 Department of Cardiovascular Medicine, Graduate School of Medicine, Chiba University, Chiba, 260-8670 Japan; 17 Department of Cardiovascular Medicine, Graduate School of Medicine, Kyoto University, Kyoto, 606-8507 Japan; 18 Department of Pharmacology, Shiga University of Medical Science, Shiga, 520-2192 Japan; 19 Department of Endocrinology, Hematology and Gerontology, Graduate School of Medicine, Chiba University, Chiba, 260-8670 Japan; 20 Department of Diabetes, Metabolism and Endocrinology, School of Medicine, International University of Health and Welfare, Chiba, 286-8686 Japan; 21 Port Square Kashiwado Clinic, Kashiwado Memorial Foundation, Chiba, 260-0025 Japan; 22 Department of Molecular Pathology, Graduate School of Medicine, Chiba University, Chiba, 260-8670 Japan; 23 Department of Laboratory Medicine & Division of Clinical Genetics, Chiba University Hospital, Chiba, 260-8677 Japan; 24 Department of Medical Technology and Sciences, School of Health Sciences at Narita, International University of Health and Welfare, Chiba, 286-8686 Japan; 25 Division of Clinical Genetics, Chiba Foundation for Health Promotion and Disease Prevention, Chiba, 261-0002 Japan; 26 Department of Frontier Surgery, Graduate School of Medicine, Chiba University, Chiba, 260-8670 Japan; 27 Department of Allergy and Rheumatology, Chiba Children’s Hospital, Chiba, 266-0007 Japan; 28 Department of Gastroenterology, Graduate School of Medicine, Chiba University, Chiba, 260-8670 Japan; 29 Department of Respirology, Graduate School of Medicine, Chiba University, Chiba, 260-8670 Japan; 30 Department of Advanced Medicine in Pulmonary Hypertension, Graduate School of Medicine, Chiba University, Chiba, 260-8670 Japan; 31 Department of Gastroenterological Surgery and Clinical Oncology, Toho University Graduate School of Medicine, Tokyo, 143-8541 Japan; 32 Division of Structural and Synthetic Biology, RIKEN Center for Life Science Technologies, Yokohama, Kanagawa 230-0045 Japan; 33 RIKEN Structural Biology Laboratory, Yokohama, Kanagawa 230-0045 Japan; 34 Celish FD Inc., Chiba, Japan; 35 Medical Project Division, Research Development Center, Fujikura Kasei Co., Saitama, 340-0203 Japan

**Keywords:** Acute ischemic stroke, Antibody biomarker, Atherosclerosis, Acute myocardial infarction, Diabetes mellitus, Chronic kidney disease

## Abstract

**Background:**

Acute ischemic stroke (AIS) is a serious cause of mortality and disability. AIS is a serious cause of mortality and disability. Early diagnosis of atherosclerosis, which is the major cause of AIS, allows therapeutic intervention before the onset, leading to prevention of AIS.

**Methods:**

Serological identification by cDNA expression cDNA libraries and the protein array method were used for the screening of antigens recognized by serum IgG antibodies in patients with atherosclerosis. Recombinant proteins or synthetic peptides derived from candidate antigens were used as antigens to compare serum IgG levels between healthy donors (HDs) and patients with atherosclerosis-related disease using the amplified luminescent proximity homogeneous assay-linked immunosorbent assay.

**Results:**

The first screening using the protein array method identified death-inducer obliterator 1 (DIDO1), forkhead box J2 (FOXJ2), and cleavage and polyadenylation specificity factor (CPSF2) as the target antigens of serum IgG antibodies in patients with AIS. Then, we prepared various antigens including glutathione S-transferase-fused DIDO1 protein as well as peptides of the amino acids 297–311 of DIDO1, 426–440 of FOXJ2, and 607–621 of CPSF2 to examine serum antibody levels. Compared with HDs, a significant increase in antibody levels of the DIDO1 protein and peptide in patients with AIS, transient ischemic attack (TIA), and chronic kidney disease (CKD) but not in those with acute myocardial infarction and diabetes mellitus (DM). Serum anti-FOXJ2 antibody levels were elevated in most patients with atherosclerosis-related diseases, whereas serum anti-CPSF2 antibody levels were associated with AIS, TIA, and DM. Receiver operating characteristic curves showed that serum DIDO1 antibody levels were highly associated with CKD, and correlation analysis revealed that serum anti-FOXJ2 antibody levels were associated with hypertension. A prospective case–control study on ischemic stroke verified that the serum antibody levels of the DIDO1 protein and DIDO1, FOXJ2, and CPSF2 peptides showed significantly higher odds ratios with a risk of AIS in patients with the highest quartile than in those with the lowest quartile, indicating that these antibody markers are useful as risk factors for AIS.

**Conclusions:**

Serum antibody levels of DIDO1, FOXJ2, and CPSF2 are useful in predicting the onset of atherosclerosis-related AIS caused by kidney failure, hypertension, and DM, respectively.

**Supplementary Information:**

The online version contains supplementary material available at 10.1186/s12916-021-02001-9.

## Background

Atherosclerosis is a serious disease and a major cause of acute ischemic stroke (AIS) and acute myocardial infarction (AMI) [1]. Diabetes mellitus (DM) and chronic kidney disease (CKD) are closely related to and accompanied by atherosclerosis [2]. As atherosclerosis progresses, atherosclerotic plaques are formed on artery walls by foam cells, which are differentiated from smooth muscle cells or macrophages [3–5]. Diagnosing atherosclerosis is important to prevent the onset of AIS and AMI because the effectiveness of treatment and therapy is limited after their onset. Thus, to date, many risk factors and biomarkers including family history, age, obesity, smoking habit, dyslipidemia, hypertension, sleep, C-reactive protein level, interleukin-6 level, troponin level, and B-type natriuretic peptide level have been reported [6, 7]; however, they are still insufficient. Genome-wide association studies on stroke have identified many genes such as *NOTCH3* [8], *CSTA* [9], and *COL3A1* [10]. However, lifestyle diseases such as stroke and atherosclerosis can be prevented by improving individuals’ lifestyles.

Recent studies have discovered that the development of autoantibodies is not limited to autoimmune diseases but is also observed in other diseases. Some examples include autoantibody markers against proteins such as p53, NY-ESO-1, and RALA for cancer [11–14]; Hsp60 for stroke [15]; insulin [16], glutamic acid decarboxylase [17], and protein tyrosine phosphatase IA-2 [18, 19] for DM, as well as phospholipid [20], apolipoprotein A1 [21, 22], oxidized low-density lipoprotein [22, 23], and heat shock proteins [22, 24] for cardiovascular disease (CVD).

Previously, we searched for antibody markers using serological identification of antigens by cDNA expression cloning (SEREX) and the protein array method, and we reported on autoantibodies against Trop2/TACSTD2 [25], TRIM21 [26], Makorin 1 [27], and ECSA [28], for esophageal squamous cell carcinoma; FIR/PUF60 for colon cancer [29]; SH3GL1 [30] and filamin C [31] for glioma; EP300-interacting inhibitor of differentiation 3 for nonfunctional pancreatic neuroendocrine tumors [32]; proline-rich 13 for ulcerative colitis [33]; talin-1 for multiple sclerosis [34]; PSMA7 for amyotrophic lateral sclerosis [35]; NBL1/DAN [36] and SNX16 [37] for obstructive sleep apnea (OSA); and EXD2 for chronic thromboembolic pulmonary hypertension (CTEPH) [38]. We also reported on autoantibody markers for atherosclerosis-related diseases, e.g., RPA2 [39], PDCD11 [40], MMP1 [41], and DNAJC2 [42] for AIS; ASXL2 [43] for atherosclerosis; and nardilysin for acute coronary syndrome [44]. Here, we report on antibodies against death-inducer obliterator 1 (DIDO1), forkhead box J2 (FOXJ2), and cleavage and polyadenylation specificity factor (CPSF2) peptides, which are highly associated with AIS and could be useful as predictive markers.

## Methods

The data that support the findings of this study are available from the corresponding author upon reasonable request.

### Patient and controls

This study was approved by the Local Ethical Review Board of the Chiba University Graduate School of Medicine (Chiba, Japan) as well as the review boards of the cooperating hospitals or institutes. Sera were collected from patients who had provided informed consent. Each serum sample was centrifuged at 3000*g* for 10 min, and supernatant was stored at − 80°C until use. Repeated freezing and thawing of samples was avoided.

Serum samples from patients with DM, ulcerative colitis, CTEPH, pulmonary arterial hypertension (PAH), and OSA were obtained from Chiba University Hospital, and samples collected from patients with AIS, transient ischemic attack (TIA), asymptomatic cerebral infarction (asympt-CI), chronic-phase CI (cCI), and deep and subcortical white matter hyperintensity (DSWMH) were obtained from Chiba Prefectural Sawara Hospital, Chiba Rosai Hospital, Chiba Aoba Municipal Hospital, and Chiba Medical Center. The stroke subtype of each patient was also determined according to the criteria of the Trial of Org 10172 in Acute Stroke Treatment classification system [45]. In this analysis, large-artery atherosclerosis or small-artery occlusion (lacune) were included as AIS or cerebral infarction.

Serum samples from patients with AIS used in the preliminary screening were provided by BioBank Japan. Serum samples from patients with AMI were obtained from Kyoto University Hospital [44]. Serum samples associated with AIS, TIA, and AMI were obtained within 2 weeks after disease onset. Samples collected from patients with CKD were obtained from the Kumamoto cohort [46, 47], whereas those collected from patients with colorectal carcinoma, esophageal squamous cell carcinoma, gastric cancer, breast cancer, and pancreatic cancer were obtained from the Department of Frontier Surgery, Chiba University Hospital. Serum samples from patients with Sjögren’s syndrome were obtained from Chiba Children’s Hospital. Serum samples from patients with rheumatoid arthritis and systemic lupus erythematosus (SLE) were obtained from the National Hospital Organization, Shimoshizu Hospital, and Chiba East Hospital [48]. Serum samples from healthy donors (HDs) were obtained from Chiba University, Port Square Kashiwado Clinic, Higashi Funabashi Hospital, and Chiba Prefectural Sawara Hospital. For comparisons with TIA and AIS, serum samples from HDs were selected from patients who exhibited no abnormalities on cranial magnetic resonance imaging.

### ProtoArray^®^ screening

The first screening was performed using ProtoArray^®^ Human Protein Microarrays v. 4.0 (Thermo Fisher Scientific, Waltham, MA), which were loaded with 9480 proteins species as described previously [33, 38, 48]. In total, 30 serum samples (15 each from HDs and patients with atherosclerosis) were used to detect antigens specifically recognized by IgG antibodies in sera. Results were analyzed using the Prospector software (Thermo Fisher Scientific), which is based on M-statistics. When comparing the two groups, a cutoff for positivity was calculated for each protein using M-statistics. For both groups, the proportion of subjects with an immune response above the cutoff value was counted, and a *P* value representing the significance of the difference between both groups was calculated as described [49].

### Expression and purification of the DIDO1 protein

Total RNA was isolated from human U2OS osteosarcoma cells using the High Pure RNA Isolation Kit (Roche, Basel, Switzerland), and cDNA was synthesized using the SuperScript III First-Strand Synthesis System for RT-PCR (Thermo Fisher Scientific). The amino-terminal (amino acids 1–275) and carboxy-terminal half (amino acids 271–545) of the coding sequences of *DIDO1* cDNA were amplified via PCR using Pyrobest DNA polymerase (Takara Bio Inc., Shiga, Japan) and cloned at the *Eco*RI/*Sal*I site of pGEX-4 T-3 (GE Healthcare Life Sciences, Pittsburgh, PA), followed by confirmation by DNA sequencing. Expression of the cDNA product was induced by treating pGEX-4 T-3-*DIDO1*-transformed *Escherichia coli* (*E. coli*) with 0.1 mM isopropyl-β-D-thiogalactoside at 25°C for 4 h; the cells were subsequently lysed in BugBuster® Master Mix (Merck Millipore, Darmstadt, Germany). Then, glutathione S-transferase (GST)-tagged DIDO1 protein was purified by glutathione-Sepharose (GE Healthcare Life Sciences) column chromatography according to the manufacturer’s instructions and dialyzed against phosphate-buffered saline (PBS) as described previously [34–37, 39–43].

### Western blotting

GST-tagged amino-terminal (amino acids 1–275) and carboxy-terminal half (amino acids 271–545) DIDO1 proteins were designated as DIDO1_N_ and DIDO1_C_, respectively, and purified as described above. GST–FOXJ2 and GST–CPSF2 were purchased from Abnova (Taipei, Taiwan). GST and GST fusion proteins (0.3 μg) were separated via sodium dodecyl sulfate–polyacrylamide gel electrophoresis and electrically transferred onto nitrocellulose membranes (Advantec, Tokyo, Japan). The membranes were blocked using a blocking solution [0.5% skim milk powder in a buffer comprising 20 mM Tris-HCl (pH 7.6), 137 mM NaCl, and 0.1% Tween 20], and the blotted proteins were probed with primary antibodies including anti-GST (goat) (Rockland, Gilbertsville, PA), anti-DIDO1 (rabbit) (Aviva Systems Biology, San Diego, CA), or anti-FOXJ2 (rabbit) (Thermo Fisher Scientific), anti-CPSF2 (rabbit) (GeneTex, Irvine, CA) or from sera from HDs (#30017) or patients with TIA (#07060, #07175, and #07207) or AIS (#07115, #07581, and #07684). After incubation with horseradish peroxidase-conjugated secondary antibodies (anti-goat IgG, anti-rabbit IgG, and antihuman IgG; Santa Cruz Biotechnology, Santa Cruz, CA), immunoreactivity was determined with Immobilon™ Western HRP Substrate (Merck KGaA, Darmstadt, Germany) as previously described [25–30, 39–43].

### Epitope prediction and peptide synthesis

Possible epitope sites in the CPSF2 and FOXJ2 proteins were predicted using the ProPred program (http://www.imtech.res.in/raghava/propred/) as described previously [38, 48]. The following amino acid sequences were designed:

- bCPSF3-165: biotin-FMIEIAGVKLLYTGD
- bCPSF3-298: biotin-NINNPFVFKHISNLK
- bCPSF3-545: biotin-KPALKVFKNITVIQE
- bCPSF2-607: biotin-QVRLKDSLVSSLQFC
- bCPSF2-712: biotin-QSVFMNEPRLSDFKQ
- bFOXJ2-426: biotin-KMVNRLNWSSIEQSQ

### Peptide array method

The epitopes in the DIDO1 protein were screened comprehensively throughout the full-length DIDO1 protein using the peptide array method, in which we designed 83 peptides of 14mer derived from the DIDO1 protein. These peptides were synthesized onto cellulose membranes using Fmoc amino acids (Auto-Spot Robot ASP222; ABIMED Analysen-Technik GmbH, Langenfeld, Germany) as described previously [50]. The membranes were washed five times with PBS containing 1% (w/v) bovine serum albumin, 0.05% Tween 20, and 0.05% NaN_3_ (PBS-T-BSA) for 30 min each and then incubated with a 1:200 dilution of sera of HDs or patients with AIS for 18 h. The membranes were subsequently washed five times with PBS-T-BSA and treated with a 1:10,000 dilution of FITC-conjugated goat antihuman IgG (Jackson ImmunoResearch, West Grove, PA) for 1 h. After washing, the fluorescence levels of peptide spots were detected using the Typhoon 9400 Imager (GE Healthcare Life Sciences) with a 488-nm/520-nm filter, as described previously [30, 48, 51].

### Peptide synthesis

N-terminal biotinylated 15-mer peptide of amino acids 426–440 derived from FOXJ2 (designated as bFOXJ2-426), N-terminal biotinylated 15-mer peptide of amino acids 607–621 derived from CPSF2 (designated as bCPSF2-607), and N-terminal biotinylated 18-mer peptide of amino acids 297–314 derived from DIDO1 (designated as bDIDO1-297) were purchased from Eurofins Genomics (Tokyo, Japan). Their amino acid sequences and purity were as follows:

- bFOXJ2-426: biotin-KMVNRLNWSSIEQSQ (94.9%)
- bCPSF2-607: biotin-QVRLKDSLVSSLQFC (99.2%)
- bDIDO1-297: biotin-AMAASKKTAPPGSAVGKQ (98.4%)

### Amplified luminescent proximity homogeneous assay-linked immunosorbent assay (AlphaLISA)

AlphaLISA was performed in 384-well microtiter plates (white opaque OptiPlate™; PerkinElmer, Waltham, MA) containing either 2.5 μL of 1:100 diluted serum with 2.5 μL of GST or GST–DIDO1 protein (10 μg/mL) or biotinylated peptides (bDIDO1-297, bFOXJ2-426, and bCPSF2-607; 400 ng/mL) in AlphaLISA buffer (25 mM HEPES, pH 7.4; 0.1% casein, 0.5% Triton X-100, 1 mg/mL Dextran 500, and 0.05% ProClin 300). The reaction mixture was incubated at room temperature for 6–8 h, after which antihuman IgG-conjugated acceptor beads (2.5 μL at 40 μg/mL) and glutathione- or streptavidin-conjugated donor beads (2.5 μL at 40 μg/mL) were added and incubated, followed by another incubation at room temperature in the dark for 1–14 days. Chemical emissions were read on an EnSpire Alpha microplate reader (PerkinElmer) as described previously [32–38, 40–43, 48, 51]. Specific reactions were calculated by subtracting the Alpha counts of GST control and buffer control without antigenic peptides from the counts of GST-fusion proteins and biotinylated peptides, respectively.

### Immunohistochemical staining

Tissue samples were obtained from surgically resected carotid atherosclerotic plaques. Paraffin-embedded vascular tissues were sectioned and then dewaxed using graded alcohol and xylene. After antigen retrieval at 98°C for 40 min in 10 mM citrate buffer (pH 6.0), endogenous peroxidase was blocked using 3% hydrogen peroxide in methanol for 30 min. Then, all sections were washed three times with a wash buffer (S3006; Agilent, Santa Clara, CA) for 5 min each and incubated for 1 h with antihuman DIDO1 antibody (rabbit; Aviva Systems Biology), anti-FOXJ2 antibody (rabbit; Thermo Fisher Scientific), anti-CPSF2 antibody (rabbit; GeneTex), anti-DHPS antibody (rabbit; Proteintech, Rosemont, IL), anti-vimentin antibody (mouse; Agilent), anti-smooth muscle actin antibody (mouse; Agilent), anti-CD31 antibody (mouse; Agilent), anti-CD68 antibody (mouse; Agilent), and anti-CD34 antibody (mouse; Agilent) at 2 μg/mL at 37°C for 60 min. Subsequently, the sections were washed three times with a wash buffer (S3006) for 5 min each and then incubated with horseradish peroxidase-conjugated anti-rabbit/anti-mouse secondary antibodies (EnVision™ Detection System:, K5007; Agilent) at 37°C for 60 min. The bound antibodies were visualized with chromogen diaminobenzidine in 3% hydrogen peroxidase. Finally, the sections were counterstained with hematoxylin, dehydrated, and mounted on glass slides as described in the literature [25, 28, 39].

### Nested case–control study

A nested case–cohort study was conducted using the abovementioned AlphaLISA detection antibody levels. This study was nested within the Japan Public Health Center (JPHC)-based Prospective Study [52, 53], which involved approximately 30,000 Japanese individuals aged 40–69 years at a baseline period of 1990–1994 whose plasma samples were stored. Serum DIDO1, bDIDO1-297, bFOXJ2-426, and bCPSF2-607 antibody levels were measured in 202 cases of incidental AIS in the cohort developed between the baseline and 2008 as well as in 202 controls whose sex, age (within 2 years), date of blood sampling (within 3 months), time since last meal (within 4 h), and study location (Public Health Center area) were matched with those of the cases. We used a conditional logistic regression model to estimate odds ratios and 95% confidence intervals (CIs) for AIS with respect to serum antibody levels of the DIDO1 protein and DIDO1, FOXJ2, and CPSF2 peptides.

### Statistical analysis

Mann–Whitney *U* test, Student’s *t* test, and Kruskal–Wallis test were used to determine the significance of the differences between two groups or among multiple groups. Correlations were calculated using Spearman’s correlation analysis. All statistical analyses were performed using GraphPad Prism 5 (GraphPad Software, La Jolla, CA). The predictive values of putative disease markers were assessed using a receiver operating characteristic (ROC) curve analysis, and cutoff values were set to maximize the sums of sensitivity and specificity. All tests were two tailed, and *P* values of < 0.05 were considered statistically significant.

## Results

### Recognition of DIDO1, CPSF2, and FOXJ2 via serum igg antibodies of patients with atherosclerosis

The first screening for AIS biomarkers was performed using SEREX and the protein array method. After the second screening using serum samples from HDs obtained from Chiba University Hospital and serum samples from patients with AIS obtained from BioBank Japan, we identified 74 antibody markers for AIS, some of which have been reported previously (Table 1). Preliminary validation tests using serum samples from patients with AIS obtained from BioBank Japan showed that the antibody levels against these three antigens, DIDO1, FOXJ2, and CPSF2, were reproducibly and significantly higher in AIS sera than control HD sera. Thus, we focused on three antibody markers highly associated with AIS.

**Table 1:** List of antibody biomarkers for atherosclerosis

Abbreviated name	Accession number	Full name	Screening method	Reference
DIDO1	BC000770.1	Death inducer obliterator-1	Protein array	This report
CPSF2	NM_017437.1	Cleavage and polyadenylation specific factor 2, 100 kDa	Protein array	This report
FOXJ2	NM_018416.2	Forkhead box J2	Protein array	This report
ACTR3B	NM_020445.6	ARP3 actin-related protein 3 homolog B	SEREX	[40]
ADAMTS7	NM_014272.3	ADAM metallopeptidase with thrombospondin type 1 motif, 7	SEREX	
AR141352	NM_133494	NIMA (never in mitosis gene a)-related kinase 7	SEREX	
ASXL2	NM_018263.6	Additional sex combs-like 2	SEREX	[43]
ATP2B4	NM_001001396.2	ATPase, Ca^++^ transporting, plasma membrane 4	Protein array	[54]
BAZ1B	NM_032408	Bromodomain adjacent to zinc finger domain, 1B	SEREX	
BMP1	NM_006129.4	Bone morphogenetic protein 1	SEREX	[39, 54]
CBX1	NM_001127228	Chromobox homolog 1	SEREX	[41]
CBX5	NM_012117	Chromobox homolog 5	SEREX	[41]
CCNG2	NM_004354.3	Cyclin G2	Protein array	[48]
CEP290	NM_014684	Centrosomal protein 290 kDa	SEREX	
CLDND1	NM_001040181	Claudin domain containing 1	Protein array	[48]
COPE	CR456886	Coatomer protein complex subunit epsilon	SEREX	[36]
CRIM1	NM_016441.2	Cysteine-rich transmembrane BMP regulator 1 (chordin-like)	SEREX	
CTNNA1	NM_001903.5	Catenin alpha 1	SEREX	[40]
CTNND1	NM_001085458	Catenin delta 1	Protein array	[48]
DEF8	NM_207514	Differentially expressed in FDCP 8 homolog (mouse)	SEREX	
DHPS	NM_001930	Deoxyhypusine synthase	Protein array	[55]
DNAJA1	NM_001539	DnaJ heat shock protein family (Hsp40) member A1	SEREX	[42]
DNAJC2	NM_014377	DnaJ heat shock protein family (Hsp40) member C2	SEREX	[42]
DST	NM_015548	Dystonin	SEREX	
EEF1A1	NM_001402.5	Eukaryotic translation Elongation factor 1 alpha 1	SEREX	[56]
EEF1G	NM_001404.4	Eukaryotic translation elongation factor 1 gamma	SEREX	
EIF2A	NM_032025.3	Eukaryotic translation initiation factor 2A, 65 kDa	SEREX	
FER1L3	NM_133337	Myoferlin	SEREX	
GOPC	NM_001017408	Golgi associated PDZ and coiled-coil motif containing	SEREX	
H3F3B	NM_005324	H3 histone, family 3B	SEREX	
HM13	AF483215	Histocompatibility (minor) 13	SEREX	
HSPA8	NM_006597	Heat shock 70 kDa protein 8	SEREX	
HSPB1	NM_001540.3	Heat shock 27 kDa protein 1	SEREX	
KIAA0020	NM_014878	KIAA0020	SEREX	[56]
LGALS9	NM_009587	Galectin 9	SEREX	[39]
LRPAP1	NM_002337	Low-density lipolipoprotein receptor–related protein–associated protein 1	SEREX	[57]
MAGT1	NM_032121.5	Magnesium transporter 1	SEREX	
MMP1	NM_002421	Metalloproteinase 1	SEREX	[41]
MYBBP1A	NM_001105538	MYB binding protein 1a	Protein array	[48]
NAV2	NM_145117.4	Neuron navigator 2	SEREX	
PARC	NM_015089	p53-associated parkin-like cytoplasmic protein	SEREX	
PDCD11	NM_014976.2	Programmed cell death 11	SEREX	[40, 58]
PFKFB3	NM_004566	6-Phosphofructo-2-kinase/Fructose-2,6-biphosphatase 3	SEREX	
PHF20	NM_016436	PHD finger protein 20	SEREX	
PPP1R15A	NM_014330	Protein phosphatase 1 regulatory subunit 15A	SEREX	[39, 59]
PRCP	NM_005040.1	Prolylcarboxypeptidase	Protein array	[60]
PSAP	NM_002778	Prosaposin	SEREX	
RANBP2L1	NM_005054	RAN binding protein 2-like 1	SEREX	
RBCK1	NM_031229	RanBP-type and C3HC4-type zinc finger containing 1	SEREX	
RBPJ	NM_005349	Recombination signal binding protein for immunoglobulin kappa J region	SEREX	
ROCK1	NM_005406	Rho-associated, coiled-coil containing protein kinase 1	SEREX	
RPA1	NM_002945	Replication protein A1	SEREX	
RPA2	NM_002946	Replication protein A2	SEREX	[39]
RPL3 R	NM_000967	Ribosomal protein L3t	SEREX	
SC65	BC007942	Synaptonemal complex protein SC65	SEREX	[39]
SH3BP5	NM_004844	SH3 domain-binding protein 5	Protein array	[51]
SMARCA4	NM_001128847	SWI/SNF related, matrix associated, actin dependent regulator of chromatin, subfamily a, member 4	SEREX	
SNX16	NM_022133.4	Sorting Nexins 16	SEREX	[37]
SOSTDC1	NM_015464	Sclerostin domain containing 1	Protein array	[48]
SPARC	NM_003118	Secreted protein acidic and cysteine-rich	SEREX	
SPOCK1	NM_004598	SPARC (osteonectin), cwcv and kazal like domains proteoglycan 1	SEREX	[56]
TBC1D2	NM_001267571	TBC1 domain family, member 2	SEREX	
TBC1D4	NM_014832	TBC1 domain family, member 4	SEREX	
TEX261	NM_144582	Testis expressed 261	SEREX	
TFAM	NM_003201	Transcription factor A, mitochondrial	Protein array	[48]
THBS1	NM_003246	Thrombospondin 1	SEREX	
TMEFF1	NM_003692	Transmembrane protein with EGF-like and two follistatin-like domains 1	SEREX	
TOP3B	NM_003935	DNA topoisomerase III beta	Protein array	[48]
TUBB2C	NM_006088	Tubulin, beta 2C	SEREX	[56]
TYMS	NM_001071	Thymidylate synthetase	SEREX	
WDR36	NM_139281.2	T cell activation WD repeat protein	SEREX	[39]
XPO1	NM_003400.3	Exportin 1	SEREX	
XRCC4	NM_022406	X-ray repair cross complementing 4	SEREX	
ZFP36L1	NM_004926	ZFP36 ring finger protein like 1	SEREX	

The results of ProtoArray^®^ loaded with 9480 protein species showed that DIDO1 (accession no. BC000770.1) antibodies were observed in 4 out of 5 serum samples from patients with atherosclerosis and 1 out of 5 serum samples from HDs. FOXJ2 (accession no. NM_018416.2) antibodies were found to react with antibodies in 7 out of 15 serum samples from patients with atherosclerosis and none of the 15 serum samples from HDs. CPSF2 (100 kDa; accession no. NM_017437.1) antibodies reacted with antibodies in 5 out of 10 serum samples from patients with atherosclerosis and 2 out of 10 serum samples from HDs. Subsequently, GST fusion proteins that contained DIDO1_N_ or DIDO1_C_ were expressed in *E*. *coli* and purified via affinity chromatography. In addition, 5 predicted epitopes of CPSF2 and 1 of FOXJ2 were prepared, and the following preliminary experiments showed that serum bFOXJ2-426 and bCPSF2-607 antibody levels more highly reacted with serum antibodies in patients with AIS than with those in HDs. To examine epitopes in the DIDO1 protein recognized by serum antibodies, we synthesized a peptide array [30, 48, 51] loaded with 83 species of 14-mer peptides derived from the DIDO1 protein. bDIDO1-297, which was most closely associated with AIS, was also used as an antigen to evaluate serum antibody levels.

### Presence of serum antibodies against purified proteins in patients with TIA or AIS

**Fig. 1:**
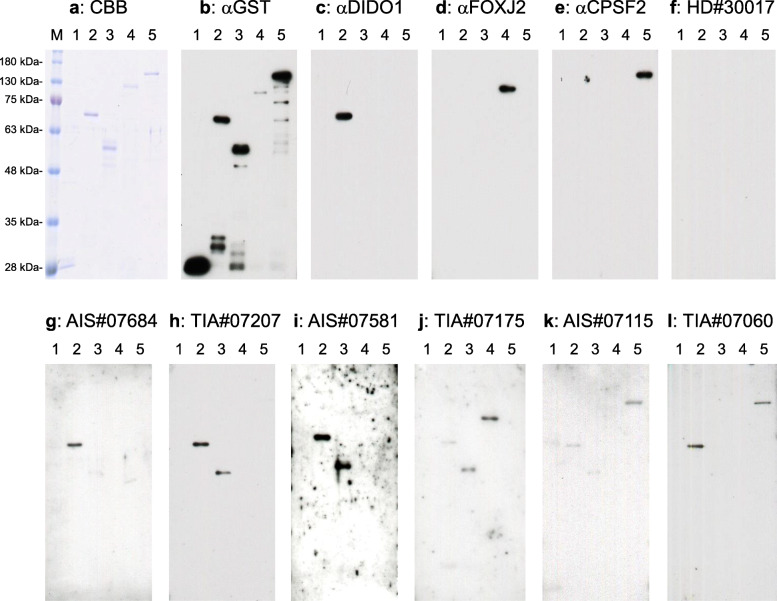
Presence of antibodies against DIDO1, FOXJ2, and CPSF2 in sera from a healthy donor (an HD) or a patient with transient ischemic attack (TIA) or acute ischemic stroke (AIS). Purified proteins of glutathione S-transferase (GST) (lane 1), GST–DIDO1 (1-275) (lane 2), GST–DIDO1 (271-545) (lane 3), GST–CPSF2 (lane 4), and GST–FOXJ2 (lane 5) were separated through sodium dodecyl sulfate-polyacrylamide gel electrophoresis, followed by western blotting analysis using anti-GST (**b**), anti-DIDO1 (**c**), anti-FOXJ2 (**d**), anti-CPSF2 (**e**), and sera from HD-#30017 (**f**), patients with AIS-#07684 (**g**), AIS-#07581 (**i**), and AIS-#07115 (**k**), and those with TIA-#07207 (**h**), TIA-#07175 (**j**), and TIA-#07060 (**l**). The Coomassie brilliant blue (CBB) staining profile is also shown in **a**. M, molecular weight marker

We then confirmed the presence of antibodies against the GST fusion proteins of DIDO1_N_, DIDO1_C_, FOXJ2, and CPSF2 in serum samples from patients with TIA or AIS via Western blotting. GST, GST–DIDO1_N_, GST–DIDO1_C_, GST–FOXJ2, and GST–CPSF2 were recognized by the anti-GST antibody as reactions of 26-, 70-, 57-, 95-, and 110-kDa proteins, respectively (Fig. 1). GST–DIDO1_N_, GST–FOXJ2, and GST–CPSF2 were recognized by each specific commercial antibody. GST–DIDO1_N_ and GST–DIDO1_C_ (but not GST) reacted with antibodies in serum samples from patients with TIA-#07207, AIS-#07684, TIA-#07175, and AIS-#07115, whereas the serum antibodies of patients with AIS-#07684 and TIA-#07060 recognized GST–DIDO1_N_ but not GST–DIDO1_C_. GST–CPSF2 reacted with antibodies in serum sample from a patient with TIA-#07175, and GST–FOXJ2 reacted with antibodies in serum sample from patients with AIS-#07115 and TIA-#07060. None of these antigenic proteins were recognized by serum IgG in patients with HD-#30017. As such, the reactivity of GST fusion antigenic proteins with serum antibodies may be primarily attributed to the antigenic protein regions but not to the GST domain. GST–DIDO1_N_ was recognized by most, if not all, serum samples from patients with AIS and TIA. Thus, in the following experiments, GST–DIDO1_N_, not GST–DIDO1_C_, was used for the measurement of antibody levels.

### Elevation of serum DIDO1-Ab, FOXJ2-Ab, and CPSF2-Ab levels in patients with TIA or AIS

**Fig. 2:**
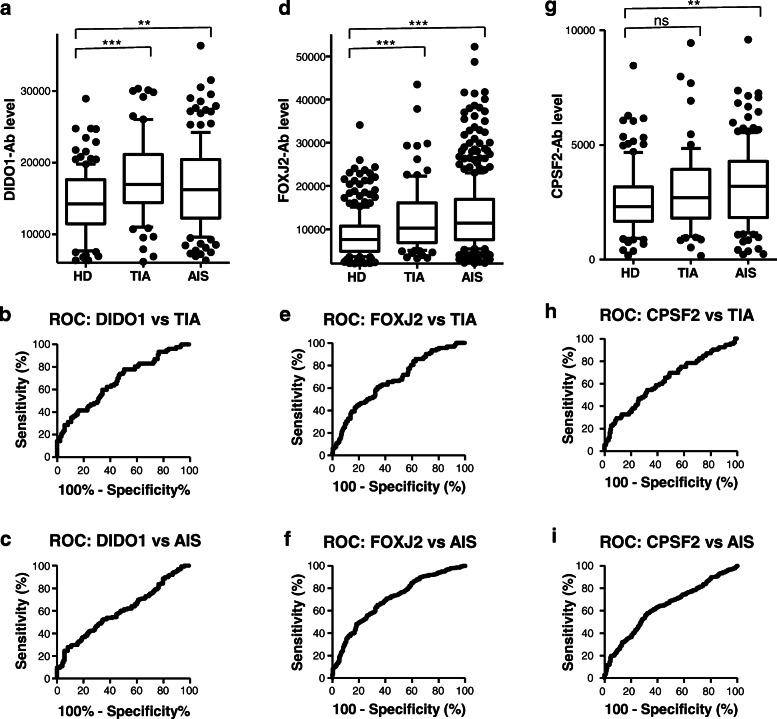
Comparison of serum DIDO1-, CPSF2-, and FOXJ2-Ab levels between HDs and patients with TIA or AIS. GST-DIDO1:1-275 (**a**), biotinylated FOXJ2 peptide (bFOXJ2-426) (**d**), and biotinylated CPSF2 peptide (bCPSF2-607) (**g**) were used as the antigens. The AlphaLISA-determined serum antibody levels after subtraction of the levels against those of control GST are shown as box-whisker plots displaying the 10th, 20th, 50th, 80th, and 90th percentiles. *P* values were calculated by the Kruskal–Wallis test. ^*^P < 0.05, ^**^P < 0.01, ^***^P < 0.001. The serum numbers of HDs, TIA, and AIS were 285, 92, and 464, respectively. The other information including total (male/female) numbers, average values, SDs, cutoff values, positive numbers, positive rates (%), and *P* values is summarized and shown in Table 2. Receiver operating characteristic curve (ROC) analysis was performed to assess the ability of DIDO1-Abs (**b**, **c**), FOXJ2-Abs (**e**, **f**), and CPSF2-Abs (**h**, **i**) to detect TIA and AIS. The numbers in the figures indicate the cutoff values for marker levels, and the numbers in parentheses indicate the sensitivity (left) and specificity (right). The areas under the curve (AUC), and 95% confidence intervals (CIs) are also shown in Table 5

**Fig. 3:**
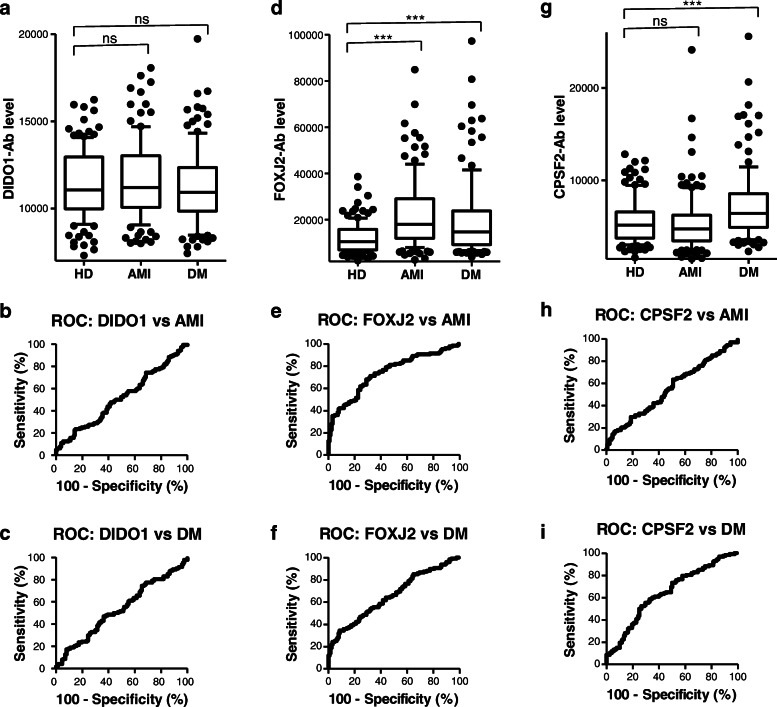
Comparison of serum DIDO1-Abs, FOXJ2-Abs, and CPSF2-Abs levels between HDs and patients with acute myocardial infarction (AMI) and diabetes mellitus (DM). GST-DIDO1:1-275 (**a**), bFOXJ2-426 (**d**), and bCPSF2-607 (**g**) were used as antigens. Serum antibody levels in HDs and patients with AMI and type 2 DM were determined using AlphaLISA and are shown as box-whisker plots, as described in the legend of Fig. 2. The same results are summarized in Table 3. Responses to DIDO1-Abs (**b**, **c**), FOXJ2-Abs (**e**, **f**), and CPSF2-Abs (**h**, **i**) were also evaluated using ROC analysis, and summarized in Table 5

We then examined the levels of anti-DIDO1_N_ protein, anti-FOXJ2 peptide (bFOXJ2-426), and anti-CPSF2 peptide (bCPSF2-607) antibodies (abbreviated as DIDO1-Ab, FOXJ2-Ab, and CPSF2-Ab, respectively) in serum samples from patients with TIA or AIS. Serum samples from HDs were obtained from the Port Square Kashiwado Clinic and compared with those from patients with TIA and AIS obtained from Chiba Prefectural Sawara Hospital, Chiba Rosai Hospital, Chiba Aoba Municipal Hospital, and Chiba Medical Center. AlphaLISA demonstrated that the serum levels of DIDO1-Ab, FOXJ2-Ab, and CPSF2-Ab were significantly higher in patients with AIS than in HDs (Fig. 2a, g and 3d). DIDO1-Abs and FOXJ2-Abs but not CPSF2-Abs were also elevated in patients with TIA as compared with those in HDs. At a cutoff value of the mean HD value plus 2 standard deviation (SD), the DIDO1-Ab positive rate in HDs and patients with TIA, AIS, and cCI was 6.7%, 15.2%, 17.5%, and 15.4%, respectively (Table 2). Their FOXJ2-Ab and CPSF2-Ab positive rates were 5.6%, 14.1%, 19.6%, and 20.0%, respectively, and 4.2%, 16.3%, 14.9%, and 21.5%, respectively.

**Table 2:** Comparison of the serum antibody levels of HDs versus those of patients with transient ischemic attack (TIA) or acute ischemic stroke (AIS)

Sample information		HD	TIA	AIS
Total sample number		285	92	464
Male/female		188/97	55/37	271/193
Age (average ± SD)		52.3 ± 11.7	70.2 ± 11.6	75.5 ± 11.5
Alpha analysis (antibody level)		DIDO1-Ab	FOXJ2-Ab	CPSF2-Ab
HD	Average	4736	8568	2515
	SD	3179	5103	1061
	Cutoff value	11,095	18,774	4638
	Positive no.	19	16	12
	Positive (%)	6.7%	5.6%	4.2%
TIA	Average	6950	12,390	3792
	SD	5251	7533	4280
	Positive no.	14	13	15
	Positive (%)	**15.2%**	**14.1%**	**16.3%**
	*P* (TIA vs HD)	**0.0002**	**< 0.0001**	**0.0056**
AIS	Average	7309	13,255	3291
	SD	5415	8042	2396
	Positive no.	81	91	69
	Positive (%)	**17.5%**	**19.6%**	**14.9%**
	*P* (AIS vs HD)	**< 0.0001**	**< 0.0001**	**< 0.0001**

The upper panel indicates the numbers of all samples and samples from males and females as well as the ages (average ± SD). The lower panel summarizes the serum antibody levels (alpha luminescent photon count) examined by AlphaLISA. Purified DIDO1 (amino acids 1-275)-glutathione S-transferase (GST) protein and synthetic peptides, bFOXJ2-426 and bCPSF2-607, were used as antigens. The cutoff values were determined as the average HD values plus two SDs, and positive samples for which the Alpha counts exceeded the cutoff value were scored. *P* values were calculated using the Kruskal–Wallis test. *P* values lower than 0.05 and positive rates higher than 10% are marked in bold. Box-whisker plots of the same results are shown in Fig. 2a, d, and g

The serum levels of anti-bDIDO1-297 peptide antibodies (DIDO1pep-Abs) were also higher in patients with TIA and AIS than in HDs (Supplementary Figure S1).

### Elevation of serum DIDO1, FOXJ2, and CPSF2 antibody levels in patients with AMI or DM

We then examined DIDO1-Ab, FOXJ2-Ab, and CPSF2-Ab in HDs and patients with AMI and DM. Serum samples from patients with AMI were obtained from Kyoto University Hospital, those from patients with DM were obtained from Chiba University Hospital, and those from HDs were obtained from the Port Square Kashiwado Clinic. The mean age (±SD) of HDs and patients with AMI and DM was 58.29 ± 5.63, 58.20 ± 8.50, and 58.37 ± 9.11 years, respectively. A total of 128 samples each of HDs and patients with AMI and type 2 DM were assayed simultaneously using AlphaLISA on a 384-well plate. Serum DIDO1-Ab levels were not visibly different between the serum samples from HDs and those from patients with AMI or DM (Fig. 3a). However, serum FOXJ2-Ab levels were significantly higher in patients with AMI or DM than in HDs (Fig. 3d). Using cutoff values as described in the previous section, positive rates were 3.1% in HDs, 34.4% in patients with AMI, and 22.7% in those with DM (Table 3). Serum CPSF2-Ab levels were significantly higher in patients with DM (although not in those with AMI) than in HDs (Fig. 3g). The positive rate of CPSF2-Ab in patients with DM was 13.3% (Table 2).

**Table 3:** Comparison of serum DIDO1-, FOXJ2-, and CPSF2-Ab levels between HDs and patients with acute myocardial infarction (AMI) or diabetes mellitus (DM) examined by AlphaLISA

Alpha analysis (antibody level)		DIDO1-Ab	FOXJ2-Ab	CPSF2-Ab
HD	Average	11,373	12,218	5571
	SD	1939	6636	2390
	Cutoff value	15,251	25,490	10,351
	Positive no.	4	4	7
	Positive (%)	3.1%	3.1%	5.5%
AMI	Average	11,634	22,965	5343
	SD	2405	16,329	3070
	Positive no.	10	44	6
	Positive (%)	7.8%	**34.4%**	4.7%
	*P* (AMI vs HD)	0.342	**< 0.0001**	0.508
DM	Average	11,199	21,718	7232
	SD	2252	24,383	3798
	Positive no.	7	29	17
	Positive (%)	5.5%	**22.7%**	**13.3%**
	*P* (DM vs HD)	0.508	**< 0.0001**	**< 0.0001**

The antigens used were purified DIDO1-GST protein and synthetic peptides, bFOXJ2-426 and bCPSF2-607. The shown numbers are as described in Table 2; *P* values lower than 0.05 and positive rates higher than 10% are marked in bold. Box-whisker plots of the same results are shown in Fig. 3a, d, and g

### Elevation of serum DIDO1, FOXJ2, and CPSF2 antibody levels in patients with CKD

**Fig. 4:**
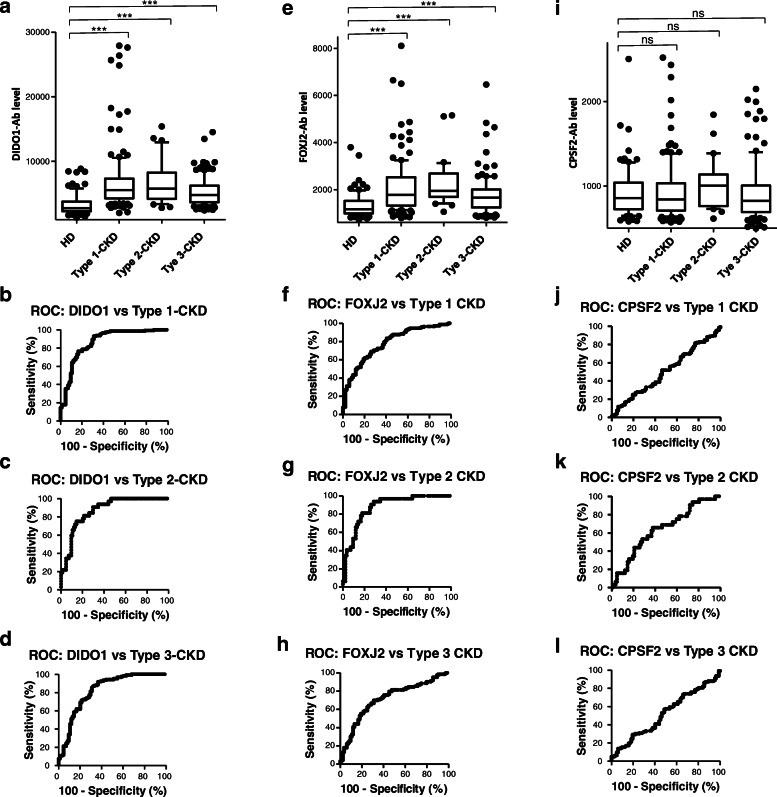
Comparison of serum DIDO1-Abs, FOXJ2-Abs, and CPSF2-Abs levels between HDs and patients with chronic kidney disease (CKD). Serum antibody levels against GST-DIDO1:1-275 protein (**a**), bFOXJ2-426 peptide (**e**), and bCPSF2-607 peptide (**i**) were compared between HDs and patients with CKD types 1, 2, and 3. The *P* values of CKD types 1, 2, and 3 versus HD controls are shown. Results are presented as described in the legend of Fig. 2. *P* values versus HD specimens are shown. The details are shown in Table 4. Responses to DIDO1-Abs (**b**–**d**), FOXJ2-Abs (**f**–**h**), and CPSF2-Abs (**j**–**l**) were also evaluated using the ROC analysis and are summarized in Table 5

Next, we examined antibody levels in serum samples from patients with CKD, which is also closely related to atherosclerosis. Patients with CKD were divided into three groups: type 1 (diabetic kidney disease), type 2 (nephrosclerosis), and type 3 (glomerulonephritis). Serum samples from patients with CKD were obtained from the Kumamoto cohort and those from HDs were obtained from Chiba University. All CKD groups had significantly higher serum DIDO1-Ab and FOXJ2-Ab levels than HDs (Fig. 4a and e). The positive rates of DIDO1-Ab in HDs and patients with type 1, type 2, and type 3 CKD were 7.3%, 43.4%, 37.5%, and 26.8%, respectively, and those of FOXJ2-Ab were 3.7%, 28.3%, 34.4%, and 13.8%, respectively (Table 4). No apparent difference was found in CPSF2-Ab levels between HDs and patients with any type of CKD (Fig. 4i, Table 4).

**Table 4:** Comparison of serum antibody levels of HDs versus those of patients with chronic kidney disease (CKD)

Sample information		HD	Type-1 CKD	Type-2 CKD	Type-3 CKD
Total sample number		82	145	32	123
Male/female		44/38	106/39	21/11	70/53
Age (average ± SD)		44.1 ± 11.2	66.0 ± 10.4	76.0 ± 9.8	62.0 ± 11.7
Alpha analysis (antibody level)		DIDO1-Ab	FOXJ2-Ab	CPSF2-Ab	
HD	Average	3166	1300	914	
	SD	1423	517	298	
	Cutoff value	6012	2334	1509	
	Positive no.	6	3	3	
	Positive rate (%)	7.3%	3.7%	3.7%	
Type 1-CKD	Average	6805	2141	939	
	SD	4675	1330	382	
	Positive no.	63	41	7	
	Positive rate (%)	**43.4%**	**28.3%**	4.8%	
	*P* (vs HD)	**< 0.0001**	**< 0.0001**	0.579	
Type 2-CKD	Average	6693	2245	1020	
	SD	3347	930	281	
	Positive no.	12	11	2	
	Positive rate (%)	**37.5%**	**34.4%**	6.3%	
	*P* (vs HD)	**< 0.0001**	**< 0.0001**	0.081	
Type 3-CKD	Average	5264	1770	936	
	SD	2161	829	421	
	Positive no.	33	17	10	
	Positive rate (%)	**26.8%**	**13.8%**	8.1%	
	*P* (vs HD)	**< 0.0001**	**< 0.0001**	0.656	

CKD types 1, 2, and 3 correspond to diabetic kidney disease, nephrosclerosis, and glomerulonephritis, respectively. The upper panel indicates the numbers of all samples and samples from males and females as well as age (average ± SD). The lower panel summarizes the serum antibody levels examined by AlphaLISA using purified DIDO1-GST protein and synthetic bCPSF2 and bFOXJ2 peptides as antigens as described in the legend of Table 2. Box-whisker plots of the same results are shown in Fig. 4a, e, and i. *P* values lower than 0.05 and positive rates higher than 10% are marked in bold

### ROC analysis

The results of the ROC analysis are shown in Figs. 2b, c, e, f, h, i, 3b, c, e, f, h, i, 4b, c, d, f, g, h, j, k, l, S1B, and S1C and summarized in Table 5, in which the area under the curve (AUC), 95% CI, cutoff value, sensitivity, specificity, and *P* value are shown. Serum anti-DIDO1 antibody levels showed markedly high AUC values against CKD. The AUCs of DIDO1-Ab versus type 1, type 2, and type 3 CKD were 0.8665, 0.8728, and 0.8227, respectively. Thus, irrespective of the CKD type, DIDO1-Ab may discriminate kidney failure. The AUCs of DIDO1-Ab versus TIA and AIS were 0.6819 and 0.6476, respectively, and similar values were observed for DIDO1pep-Ab (0.6503 and 0.6611, respectively). No significant increase above 0.6 was observed in AUCs of DIDO1-Ab versus AMI and DM.

**Table 5:** Receiver operating characteristic (ROC) analysis

	DIDO1-Ab vs TIA	DIDO1-Ab vs AIS		
AUC	0.6767	0.6023		
95% CI	0.6001–0.7533	0.5367–0.6680		
Cutoff value	14,184	19,924		
Sensitivity (%)	77.9%	27.9%		
Specificity (%)	49.6%	91.9%		
*P* value	**< 0.0001**	**0.0033**		
	DIDO1-Ab vs AMI	DIDO1-Ab vs DM		
AUC	0.5163	0.5347		
95% CI	0.4454–0.5875	0.4638–0.6057		
Cutoff value	13,519	10,700		
Sensitivity (%)	22.7%	46.9%		
Specificity (%)	85.8%	63.8%		
*P* value	0.650	0.338		
	DIDO1-Ab vs type 1 CKD	DIDO1-Ab vs type 2 CKD	DIDO1-Ab vs type 3 CKD	
AUC	**0.8665**	**0.8728**	**0.8227**	
95% CI	0.8144 to 0.9186	0.8092 to 0.9364	0.7611 to 0.8843	
Cutoff value	3375	3511	3158	
Sensitivity (%)	93.1%	90.6%	91.9%	
Specificity (%)	69.1%	70.2%	63.1%	
*P* value	**< 0.0001**	**< 0.0001**	**< 0.0001**	
	DIDO1pep-Ab vs TIA	DIDO1pep-Ab vs AIS		
AUC	0.6503	0.6611		
95% CI	0.5751–0.7256	0.6138–0.7084		
Cutoff value	4662	8413		
Sensitivity (%)	87.9%	43.9%		
Specificity (%)	38.3%	81.9%		
*P* value	**0.0003**	**< 0.0001**		
	FOXJ2-Ab vs TIA	FOXJ2-Ab vs AIS	CPSF2-Ab vs TIA	CPSF2-Ab vs AIS
AUC	0.6696	**0.7006**	0.6314	0.6369
95% CI	0.6066 to 0.7326	0.6626 to 0.7386	0.5631–0.6997	0.5970–0.6768
Cutoff value	8978	8920	2643	2644
Sensitivity (%)	60.9%	65.1%	54.4%	57.8%
Specificity (%)	66.0%	66.0%	67.7%	67.7%
*P* value	**< 0.0001**	**< 0.0001**	**0.0002**	**< 0.0001**
	FOXJ2-Ab vs AMI	FOXJ2-Ab vs DM	CPSF2-Ab vs AMI	CPSF2-Ab vs DM
AUC	**0.7418**	0.6584	0.5522	0.6464
95% CI	0.6813 to 0.8022	0.5922 to 0.7245	0.4817 to 0.6226	0.5792 to 0.7136
Cutoff value	14,437	20,978	5356	6145
Sensitivity (%)	68.0%	34.4%	63.3%	55.5%
Specificity (%)	71.1%	91.4%	49.2%	70.3%
*P* value	**< 0.0001**	**< 0.0001**	0.149	**< 0.0001**
	FOXJ2-Ab vs type 1 CKD	FOXJ2-Ab vs type 2 CKD	FOXJ2-Ab vs type 3 CKD	
AUC	**0.7812**	**0.8769**	**0.7151**	
95% CI	0.7200 to 0.8424	0.8124 to 0.9413	0.6439 to 0.7862	
Cutoff value	1236	1391	1354	
Sensitivity (%)	83.5%	93.8%	69.9%	
Specificity (%)	59.5%	71.4%	69.1%	
*P* value	**< 0.0001**	**< 0.0001**	**< 0.0001**	
	CPSF2-Ab vs Type-1 CKD	CPSF2-Ab vsType-2 CKD	CPSF2-Ab vsType-3 CKD	
AUC	0.5040	0.6387	0.5196	
95% CI	0.4262–0.5817	0.5274–0.7500	0.4395–0.5996	
Cutoff value	641.5	901	706	
Sensitivity (%)	11.7%	65.6%	29.3%	
Specificity (%)	93.9%	62.2%	80.5%	
*P* value	0.921	**0.022**	0.635	

Area under the curve (AUC), 95% CI, cutoff value, sensitivity (%), specificity (%), and *P* value of the ROC analysis are shown. Purified GST-DIDO1 protein and synthetic peptides—bDIDO1-297 (DIDO1pep), bFOXJ2-426, and bCPSF2-607—were used as antigens. *P* values lower than 0.05 and AUCs higher than 0.7 are marked in bold

AUCs of FOXJ2-Ab were > 0.65 versus TIA, AIS, AMI, DM, and CKD, among which AUC was the highest versus type 2 CKD (0.8769; Table 4). AUC versus DM was relatively low (0.6584). Thus, FOXJ2-Ab may be associated with kidney failure and atherosclerosis, but it does not primarily reflect DM. However, CPSF2-Ab was not associated with AMI or type 1/type 2/type 3 CKD. The lowest *P* values were observed versus AIS and DM, suggesting that CPSF2-Abs reflect diabetic AIS.

### Serum DIDO1-Ab, FOXJ2-Ab, and CPSF2-Ab levels in cancer

Because autologous antibodies often develop in patients with cancer [11–14], we examined serum samples from patients with colorectal carcinoma, esophageal squamous cell carcinoma, gastric cancer, breast cancer, and pancreatic cancer obtained from Chiba University Hospital. Notably, serum DIDO1-Ab and CPSF2-Ab levels were not significantly different between HDs and patients with any type of cancer (Supplementary Table S1). However, serum FOXJ2-Ab levels were significantly higher in patients with colorectal carcinoma but not in those with other types of cancer than in HDs.

### Association of serum DIDO1-Ab, FOXJ2-Ab, and CPSF2-Ab levels with autoimmune diseases

Autoantibodies may have causal effects on autoimmune diseases such as Sjögren’s syndrome, rheumatoid arthritis, SLE, and ulcerative colitis. Some of these autoimmunity-related characteristics are known to be involved in the development of atherosclerosis [61–64]. We examined antibody levels in serum samples from patients with Sjögren’s syndrome, rheumatoid arthritis, SLE, and ulcerative colitis. Serum DIDO1-Ab and FOXJ2-Ab levels were significantly higher in patients with rheumatoid arthritis and SLE (but not in those with Sjögren’s syndrome or ulcerative colitis) than in HDs (Supplementary Table S2). Serum CPSF2-Ab levels were higher in patients with rheumatoid arthritis (but not in those with Sjögren’s syndrome, SLE, or ulcerative colitis) than in HDs.

### Association of serum DIDO1-Ab, FOXJ2-Ab, and CPSF2-Ab levels with pulmonary diseases

OSA is frequently accompanied by hypertension. Serum anti-COPE was identified by SEREX screening using serum samples from patients with atherosclerosis, and its level was elevated in patients with OSA compared with in HDs [65]. Pulmonary diseases including CTEPH and PAH are distinct from hypertension but could have an inflammatory condition similar to that in hypertension (e.g., elevation of Pentraxin 3 level) [66]. Serum FOXJ2-Ab levels were higher in patients with CTEPH and PAH (but not in those with OSA) than in HDs, whereas serum DIDO1-Ab and CPSF2-Ab levels did not show any apparent difference between HDs and patients with CTEPH, PAH, or OSA (Supplementary Table S3).

### Correlation analysis

Comparative analysis of serum antibody levels and subject data was performed using 851 serum samples obtained from Chiba Prefectural Sawara Hospital including 188 serum samples from HDs, 162 from patients with DSWMH, 18 from patients with asympt-CI, 66 from patients with TIA, 351 from patients with AIS, 66 from patients with cCI, and 66 from disease controls. Other subject information is shown in Supplementary Table S4. Comparison using Mann–Whitney *U* test revealed that serum DIDO1pep-Ab, FOXJ2-Ab, and CPSF2-Ab levels were significantly higher in patients with TIA, AIS, and cCI (but not in those with DSWMH) than in HDs (Table 6, uppermost panel). Then, antibody levels were compared between males and females; those with or without DM, hypertension, CVD, and dyslipidemia; and those with or without smoking and alcohol intake habits. Hypertension was defined as a history of systolic blood pressure of > 140 mmHg, diastolic blood pressure of > 90 mmHg, or use of antihypertensive agents. Significantly higher serum DIDO1pep-Ab levels were observed in patients with hypertension, CVD, dyslipidemia, or a smoking habit (but not in those with DM) than in their control groups (Table 6, lower panels). Serum FOXJ2-Ab levels showed similar results, except that they were not correlated with dyslipidemia. Meanwhile, serum CPSF2-Ab levels were associated with DM, hypertension, and smoking habit but not with CVD or dyslipidemia. Sex and alcohol intake displayed no association with any of these three antibody levels.

**Table 6:** Correlation analysis of antibody levels against synthetic bDIDO1, bCPSF2, and bFOXJ2 peptides with data of subjects in the Sawara Hospital cohort

Present disease		HD	DSWMH	asympt-CI	TIA	AIS	cCI
Sample number		188	162	18	66	351	66
DIDO1pep-Ab level	Average	3381	3523	3481	4443	4688	4347
	SD	1660	1750	2099	2576	2740	3017
*P* value (vs HD)		–	ns	ns	**< 0.01**	**< 0.001**	**< 0.05**
FOXJ2-Ab level	Average	4627	4995	4902	5794	6298	7022
	SD	1972	2232	1854	2368	3308	5646
*P* value (vs HD)		–	ns	ns	**< 0.01**	**< 0.001**	**< 0.001**
CPSF2-Ab level	Average	7322	7571	8312	11,778	8722	10,088
	SD	3415	2942	2461	16,843	3970	4240
*P* value (vs HD)		–	ns	**< 0.05**	**< 0.01**	**< 0.001**	**< 0.001**
Sex		Male	Female				
Sample number		528	389				
DIDO1pep-Ab level	Average	4081	4038				
	SD	2493	2244				
*P* value (vs Male)			0.781				
FOXJ2-Ab level	Average	5772	5443				
	SD	3077	3084				
*P* value (vs male)			0.111				
CPSF2-Ab level	Average	8633	8420				
	SD	5493	6553				
*P* value (vs male)			0.155				
Complication		DM−	DM+				
Sample number		732	180				
DIDO1pep-Ab level	Average	4059	4047				
	SD	2469	2027				
*P* value (vs DM−)			0.949				
FOXJ2-Ab level	Average	5589	5763				
	SD	3104	2987				
*P* value (vs DM−)			0.488				
CPSF2-Ab level	Average	8319	9437				
	SD	5373	7822				
*P* value (vs DM−)			**0.015**				
Complication		HT−	HT+				
Sample number		347	565				
DIDO1pep-Ab level	Average	3830	4196				
	SD	2217	2477				
*P* value (vs HT−)			**0.021**				
FOXJ2-Ab level	Average	5093	5948				
	SD	2373	3405				
*P* value (vs HT−)			**< 0.0001**				
CPSF2-Ab level	Average	7699	9065				
	SD	6095	5804				
*P* value (vs HT−)			**< 0.0001**				
Complication		CVD−	CVD+				
Sample number		861	51				
DIDO1pep-Ab level	Average	4003	4966				
	SD	2360	2673				
*P* value (vs CVD−)			**0.015**				
FOXJ2-Ab level	Average	5559	6712				
	SD	3050	3408				
*P* value (vs CVD−)			**0.022**				
CPSF2-Ab level	Average	8499	9232				
	SD	6037	4239				
*P* value (vs CVD−)			0.142				
Complication		Lipidemia−	Lipidemia+				
Sample number		649	263				
DIDO1pep-Ab level	Average	4158	3806				
	SD	2497	2073				
*P* value (vs Lipidemia−)			**0.029**				
FOXJ2-Ab level	Average	5702	5428				
	SD	3171	2841				
*P* value (vs Lipidemia−)			0.203				
CPSF2-Ab level	Average	8146	9531				
	SD	3583	9534				
*P* value (vs Lipidemia−)			0.145				
Lifestyle		Non-smoker	Smoker				
Sample number		474	441				
DIDO1pep-Ab level	Average	3732	4425				
	SD	2037	2676				
*P* value (vs non-smoker)			**< 0.0001**				
FOXJ2-Ab level	Average	5192	6111				
	SD	2793	3309				
*P* value (vs non-smoker)			**< 0.0001**				
CPSF2-Ab level	Average	8214	8901				
	SD	6086	5801				
*P* value (vs non-smoker)			**0.002**				
Lifestyle		Alcohol−	Alcohol+				
Sample number		334	581				
DIDO1pep-Ab level	Average	4001	4103				
	SD	2236	2476				
*P* value (vs Alcohol−)			0.527				
FOXJ2-Ab level	Average	5691	5603				
	SD	3542	2793				
*P* value (vs Alcohol−)			0.698				
CPSF2-Ab level	Average	8559	8591				
	SD	6946	5341				
*P* value (vs Alcohol−)			0.361				

The subjects were divided as follows: sex (male and female); presence (+) or absence (−) of complication of DM, hypertension (HT), cardiovascular disease (CVD), or dyslipidemia, and lifestyle factors (smoking and alcohol intake habits). Antibody levels (Alpha counts) were compared using the Kruskal–Wallis test (upper panel) and the Mann–Whitney *U* test (lower panels). Sample numbers, averages, and SDs of counts as well as *P* values are shown. Significant correlations (*P* < 0.05) are marked in bold

Spearman’s rank-order correlation analysis was performed to determine the correlation between serum antibody levels of DIDO, FOXJ2, and CPSF2 peptides and subject parameters including general information such as age, body height, weight, body mass index, and degree of artery stenosis (maximum intima media thickness, max IMT). The following blood test data were also included: albumin/globulin ratio, aspartate aminotransferase, alanine amino transferase, alkaline phosphatase, lactate dehydrogenase, total bilirubin, cholinesterase, γ-glutamyl transpeptidase, total protein, albumin, blood urea nitrogen, creatinine, estimated glomerular filtration rate, uric acid, amylase, total cholesterol, high-density lipoprotein cholesterol, triglyceride, sodium, potassium, chlorine, calcium, inorganic phosphate, iron, C-reactive protein, low-density lipoprotein cholesterol, white blood cells, red blood cells, hemoglobin, hematocrit, mean corpuscular volume, mean corpuscular hemoglobin, mean corpuscular hemoglobin concentration, red cell distribution width, platelets, mean platelet volume, procalcitonin, platelet distribution width, blood sugar, and glycated hemoglobin (HbA1c).

All three antibody levels were correlated with age and max IMT but inversely correlated with height and weight and cholinesterase, total protein, and albumin levels. Serum DIDO1 and FOXJ2 antibody levels, but not serum CPSF2 antibody level, were correlated with alkaline phosphatase, white blood cell count, and mean corpuscular volume (Table 7). Blood sugar and HbA1c, which reflect DM, were not correlated with these antibody levels, except for a slight correlation (*P* = 0.195) between serum DIDO1pep-Ab level and blood sugar.

**Table 7:** Correlation analysis of serum antibody levels against synthetic bDIDO1, bCPSF2, and bFOXJ2 peptides with data on subjects in the Sawara Hospital cohort

Parameter*	Number of XY pairs	DIDO1pep-Ab		FOXJ2pep-Ab		CPSF2pep-Ab	
		*r* value**	*P* value	*r* value	*P* value	*r* value	*P* value
Age	851	0.2074	< 0.0001***	0.2688	**< 0.0001**	0.1657	**< 0.0001**
Height	844	− 0.1227	**0.0004**	− 0.1229	**0.0003**	− 0.0799	**0.0202**
Weight	848	− 0.1047	**0.0023**	− 0.1196	**0.0005**	− 0.0707	**0.0396**
BMI	843	− 0.0311	0.3679	− 0.0552	0.1098	− 0.0343	0.3197
max IMT	646	0.1908	**< 0.0001**	0.2717	**< 0.0001**	0.2161	**< 0.0001**
A/G	820	− 0.0303	0.3858	− 0.0484	0.1662	− 0.0906	**0.0094**
AST	848	0.0605	0.0782	0.0205	0.5523	− 0.0496	0.1490
ALT	847	0.0063	0.8545	− 0.0079	0.8177	− 0.0800	**0.0199**
ALP	786	0.0850	**0.0172**	0.0743	**0.0374**	0.0319	0.3716
LDH	822	0.0718	**0.0395**	0.0291	0.4046	− 0.0134	0.7017
tBil	830	− 0.0576	0.0972	− 0.0752	**0.0304**	− 0.1024	**0.0031**
CHE	646	− 0.0895	**0.0230**	− 0.1671	**< 0.0001**	− 0.0982	**0.0125**
γ-GTP	795	0.0334	0.3474	0.0240	0.4996	− 0.0028	0.9381
TP	823	− 0.0971	**0.0053**	− 0.1443	**< 0.0001**	− 0.1084	**0.0018**
Albumin	832	− 0.0757	**0.0289**	− 0.1294	**0.0002**	− 0.1358	**< 0.0001**
BUN	846	0.0179	0.6038	0.0431	0.2103	− 0.0381	0.2686
CRE	842	− 0.0090	0.7946	0.0472	0.1714	− 0.0341	0.3233
eGFR	758	0.0176	0.6284	− 0.0255	0.4835	0.0230	0.5282
UA	622	0.0336	0.4023	0.0255	0.5261	0.0050	0.9006
AMY	527	− 0.0780	0.0735	− 0.0422	0.3350	− 0.0391	0.3701
T-CHO	744	− 0.0520	0.1568	− 0.0604	0.0994	− 0.1207	**0.0010**
HDL-C	550	− 0.0458	0.2840	− 0.0521	0.2222	0.0553	0.1952
TG	589	0.0199	0.6303	0.0038	0.9274	− 0.0405	0.3261
Na	833	0.0200	0.5635	0.0233	0.5027	0.0005	0.9881
K	832	− 0.0275	0.4280	− 0.0091	0.7928	− 0.0072	0.8359
Cl	833	0.0056	0.8708	0.0470	0.1752	0.0269	0.4376
Ca	495	− 0.0210	0.6408	− 0.0815	0.0708	− 0.0405	0.3682
IP	388	− 0.0023	0.9639	− 0.0465	0.3618	0.0546	0.2836
Fe	400	− 0.0406	0.4185	− 0.0575	0.2526	− 0.0472	0.3465
CRP	617	0.1172	**0.0035**	0.0775	0.0552	0.1041	**0.0096**
LDL-C	440	− 0.0513	0.2831	− 0.0771	0.1071	− 0.1180	**0.0133**
WBC	846	0.1036	**0.0026**	0.0848	**0.0138**	0.0417	0.2262
RBC	846	− 0.0426	0.2155	− 0.0649	0.0596	− 0.0711	**0.0386**
HGB	846	− 0.0113	0.7420	− 0.0329	0.3406	− 0.0672	0.0508
HCT	846	− 0.0078	0.8214	− 0.0271	0.4317	− 0.0528	0.1249
MCV	846	0.0683	**0.0472**	0.0959	**0.0053**	0.0510	0.1387
MCH	846	0.0474	0.1681	0.0776	**0.0242**	0.0081	0.8136
MCHC	846	− 0.0149	0.6659	− 0.0253	0.4635	− 0.0617	0.0728
RDW	846	0.0489	0.1551	0.0449	0.1928	0.0529	0.1245
PLT	846	− 0.0047	0.8919	− 0.0443	0.1992	0.0128	0.7097
MPV	846	− 0.0201	0.5589	− 0.0637	0.0646	− 0.0012	0.9716
PCT	846	− 0.0030	0.9312	− 0.0568	0.0993	0.0188	0.5853
PDW	846	− 0.0151	0.6611	− 0.0587	0.0886	− 0.0109	0.7512
BS	783	0.0834	**0.0195**	0.0678	0.0581	0.0644	0.0718
HbA1c	655	− 0.0204	0.6031	− 0.0170	0.6644	− 0.0277	0.4789

*Subjects’ data used were age, height, weight, body mass index (BMI), maximum intima–media thickness (max IMT), albumin/globulin ratio (A/G), aspartate aminotransferase (AST), alanine amino transferase (ALT), alkaline phosphatase (ALP), lactate dehydrogenase (LDH), total bilirubin (tBil), cholinesterase (CHE), γ-glutamyl transpeptidase (γ-GTP), total protein (TP), albumin, blood urea nitrogen (BUN), creatinine (CRE), estimated glomerular filtration rate (eGFR), uric acid (UA), amylase (AMY), total cholesterol (T-CHO), high-density lipoprotein cholesterol (HDL-C), triglyceride (TG), sodium (Na), potassium (K), chlorine (Cl), calcium (Ca), inorganic phosphate (IP), iron (Fe), C-reactive protein (CRP), low-density lipoprotein cholesterol (LDL-C), white blood cells (WBC), red blood cells (RBC), hemoglobin (HGB), hematocrit (HCT), mean corpuscular volume (MCV), mean corpuscular hemoglobin (MCH), MCH concentration (MCHC), red cell distribution width (RDW), platelets (PLT), mean platelet volume (MPV), procalcitonin (PCT), platelet distribution width (PDW), blood sugar (BS), and glycated hemoglobin (HbA1c)

**Correlation coefficients (*r* values) and *P* values obtained through Spearman’s correlation analysis are shown

***Significant correlations (*P* < 0.05) are marked in bold

### Immunohistochemical analysis of antigenic proteins

**Fig. 5:**
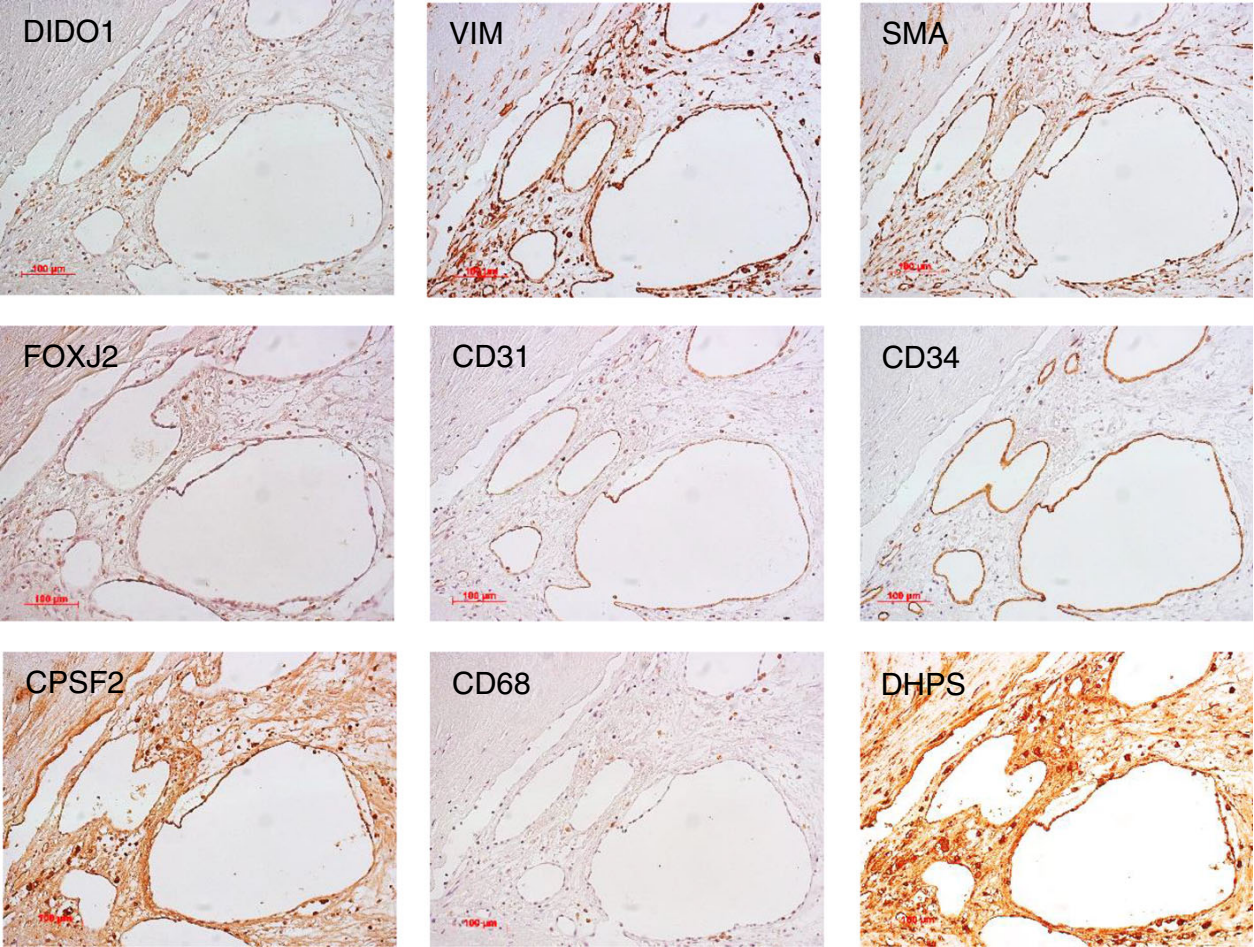
Immunohistochemical staining of antigenic marker proteins in the atherosclerotic lesions. Surgically resected carotid atherosclerotic plaques were stained using immunohistochemistry. The antibodies used were anti-DIDO1 (Aviva Systems Biology), anti-FOXJ2 (Thermo Fisher Scientific), anti-CPSF2 (GeneTex), and anti-DHPS (Proteintech) antibodies for comparison. The tissue was also stained with antibodies against smooth muscle cell marker, vimentin (VIM) and smooth muscle actin (SMA), vascular endothelial cell marker, CD31 and CD34, and macrophage marker, CD68

Assuming that autoantibodies against DIDO1, FOXJ2, and CPSF2 peptides develop in patients with atherosclerotic diseases, these antigenic proteins should be expressed at high levels in atherosclerotic lesions. As such, we also examined the expressions of antigenic proteins in surgically resected carotid atherosclerotic plaques via immunohistochemistry. The DIDO1 and CPSF2 proteins were predominantly expressed in the intima of atherosclerotic plaques, similar to the localization of vimentin and smooth muscle actin, which are markers for smooth muscle cells (Fig. 5). DHPS, reported as an atherosclerosis marker [55], was also expressed in smooth muscle cells. The expression of FOXJ2 showed a similar pattern as that of CD31- and CD34-positive vascular endothelial cells. CD68 expression in macrophages was not similar to any of the other antigen expressions (Fig. 5).

### JPHC cohort analysis

We conducted a case–control study nested within the JPHC-based Prospective Study, which involved approximately 30,000 plasma samples [52, 53]. The antibody level against the DIDO1 protein was positively and strongly associated with a risk of AIS: odds ratios (95% CIs) were 3.99 (1.93–8.23), 3.40 (1.62–7.13), and 4.02 (1.94–8.35) for those with the second, third, and highest quartiles of antibody levels, respectively, versus for those with the lowest quartile (Table 8). Likewise, the antibody levels of the DIDO1, FOXJ2, and CPSF2 peptides were positively correlated with a risk of cerebral infarction: odds ratios (95% CIs) of the highest quartile were 2.66 (1.43–4.95), 2.24 (1.27–3.95), and 2.41 (1.33–4.37), respectively. These results indicate that the antibody markers against the DIDO1 protein and DIDO1, FOXJ2, and CPSF2 peptides are useful in predicting the onset of AIS.

**Table 8:** Results of the Japan Public Health Center (JPHC) cohort samples

		DIDO1-Ab vs AIS	DIDO1pep-Ab vs AIS	FOXJ2-Ab vs AIS	CPSF2-Ab vs AIS
2nd	Matched OR	3.99	1.92	1.43	1.19
	95% CI	1.93–8.23	1.03–3.58	0.78–2.62	0.63–2.23
3rd	Matched OR	3.40	2.40	1.32	1.66
	95% CI	1.62–7.13	1.29–4.46	0.72–2.43	0.89–3.09
Max	Matched OR	4.02	2.66	2.24	2.41
	95% CI	1.94–8.35	1.43–4.95	1.27–3.95	1.33–4.37

The odds ratios (ORs) and 95% CI of the 2nd, 3rd, and the highest (max) quartiles versus the lowest quartile are shown for AIS with respect to the antibody levels of DIDO1 protein, DIDO1 peptide, FOXJ2 peptide, and CPSF2 peptide

## Discussion

### Three novel antibody markers for atherosclerosis

We performed large-scale screening using SEREX and the protein microarray method and identified 69 candidate antigenic proteins related to atherosclerosis (Table 1). In the present study, we focused on three antigens—DIDO1, FOXJ2, and CPSF2—that appeared to be of much interest in relation to AIS. The presence of antibodies against these proteins was confirmed by Western blotting (Fig. 1). We then examined epitopes and selected bDIDO1-297, bFOXJ2-426, and bCPSF2-607 as useful antigenic peptides to measure serum antibody levels. The amino-terminal half of DIDO1 was also used as an antigen. Serum antibody levels of these antigens were more elevated in patients with AIS and TIA than in HDs (Fig. 2, Supplementary Figure S1). All of bDIDO1-297, bFOXJ2-426, and bCPSF2-607 were closely correlated with max IMT (Table 7), which is a typical index of the development of atherosclerosis leading to AIS and CVD [67–70]. Thus, these serum antibodies can be markers for atherosclerosis. A case–control study nested within the JPHC-based Prospective Study showed that the three antibody markers are associated with the risk of cerebral infarction and indicated that these markers are useful in predicting the onset of cerebral infarction (Table 8). However, they had distinct characteristics.

The DIDO1 protein was first identified as a regulator of apoptosis [71]. Serum DIDO1pep-Ab levels were elevated in patients with TIA, AIS, cCI, CKD, rheumatoid arthritis, and SLE but not in those with AMI, DM, any type of cancer, or ulcerative colitis (Figs. 2, 3, and 4; Tables 2, 3 and 4; Supplementary Tables S2 and S3). In particular, the AUC values of DIDO1 versus CKD were > 0.8 (Table 5), suggesting that DIDO1-Ab reflects kidney failure basically and atherosclerosis indirectly.

FOXJ2 is a member of the forkhead family of transcription factors [72]. Serum FOXJ2-Ab levels were elevated in patients with TIA, AIS, cCI, AMI, DM, CKD, colorectal carcinoma, rheumatoid arthritis, and SLE compared with in HDs (Figs. 2, 3, and 4; Tables 2, 3, and 4; Supplementary Tables S1 and S2). Serum FOXJ2-Ab levels correlated well with hypertension (Table 6) and were elevated in patients with CTEPH and PAH (Supplementary Table S3), suggesting that these levels reflect systemic arterial hypertension and can differentiate hypertension-related diseases. In fact, hypertension is also a risk factor for colorectal carcinoma [73], and SLE is frequently associated with PAH [74].

Collagen diseases such as rheumatoid arthritis and SLE are high-risk groups of AIS and AMI [61, 62]. Serum DIDO1-Ab and FOXJ2-Ab levels were significantly associated with rheumatoid arthritis and SLE but not with Sjögren’s syndrome or ulcerative colitis (Supplementary Table S2). It is possible that DIDO1-Ab and FOXJ2-Ab are discriminant in the case of AIS of which one of the causes is a collagen disease. That is, each marker may correspond to a different cause of atherosclerosis.

*CPSF2* encodes the 100-kD subunit of CPSF, which plays a central role in the 3′ processing of pre-mRNA [75]. Serum CPSF2-Ab levels were associated primarily with AIS and DM and partly with TIA, cCI, esophageal squamous cell carcinoma, and rheumatoid arthritis but not with AMI, CKD, CVD, colorectal carcinoma, gastric cancer, breast cancer, pancreatic cancer, Sjögren’s syndrome, SLE, or ulcerative colitis (Figs. 2, 3, 4; Tables 2, 3, and 4; Supplementary Tables S1 and S2). Serum CPSF2-Ab levels were correlated with aortic hypertension (Table 6) but not with pulmonary hypertension such as CTEPH and PAH (Supplementary Table S3). Moreover, the levels correlated most closely with max IMT (Table 7), indicating that CPSF2-Ab can mainly detect DM-caused atherosclerosis leading to AIS.

### Relationship between BMP/TGF-β and atherosclerosis

Bone morphogenetic proteins (BMPs) are involved in the transforming growth factor-β (TGF-β) superfamily. It is well documented that BMP signals play important roles in the development of atherosclerosis [76, 77]. BMP-2 and BMP-4 expressions were elevated in atherosclerotic endothelium [78, 79], and plasma BMP-2 levels are elevated in patients with type 2 DM [80]. Chronic infusion of BMP-4 induces endothelial dysfunction and hypertension [81], and treatment with the BMP antagonist, matrix Gla protein, and BMP inhibitors prevents the development of ATS [82, 83]. On the other hand, the knockdown of the BMP type II receptor BMPRII accelerates ATS [84]. Therefore, BMP family members may play a subtle regulatory role in the development of ATS. It should be noted that *DIDO1* is the target gene of BMP and promotes cell attachment, migration, invasion, and apoptosis resistance in melanoma [85].

CPSF proteins interact with Smad via Smicl and potentiate TGF-β/BMP-stimulated Smad-dependent transcriptional responses [86, 87]. We previously reported the elevation of autoantibodies against SOSTDC1 and NBL1/DAN, which are the antagonists of BMP, in patients with AIS [48] and OSA [36], respectively. As such, it is possible that some, if not all, autoantibodies against TGF-β/BMP-related proteins play causal or suppressive roles in the development of atherosclerosis-related diseases.

### Involvement of marker genes in development and differentiation

*DIDO1* is the target gene of Oct4, Sox2, and Nanog; in reverse, *Nanog* and *Oct4* are the target genes of DIDO1 [88]. Thus, DIDO1 plays a key role in the self-renewal of embryonic stem cells. Futterer suggested that DIDO1 is a switchboard that regulates embryonic stem cell transition from pluripotency maintenance to differentiation [89]. During the development of atherosclerosis, smooth muscle cells differentiate into foam cells to form atheroma [90]. Highly expressed DIDO1 in intimal smooth muscle cells (Fig. 5) may have an important role in their differentiation into foam cells.

FOXJ2 expression is also regulated by Oct4 and involved in oocyte development [91]. Transient FOXJ2 transgenesis experiments have shown that FOXJ2 overexpression has a lethal effect on embryonic development from E10.5 [92]. FOXJ2 is also involved in differentiation and inhibits TGF-β1-induced epithelial–mesenchymal transition [93]. Thus, high FOXJ2 expression (Fig. 5) may affect otherwise normally functioning vascular endothelial cells.

### Relationship between atherosclerosis and cancer

BMP-induced DIDO1 promotes cell attachment, migration, invasion, and apoptosis resistance in melanoma [85]. Serum FOXJ2-Ab levels, which correlated well with hypertension, were elevated in patients with colorectal carcinoma (*P* < 0.001) but not in those with esophageal squamous cell carcinoma, gastric cancer, breast cancer, or pancreatic cancer (Supplementary Table S1). This is consistent with the report that hypertension is also a risk factor for colorectal carcinoma but not for esophageal squamous cell carcinoma or gastric cancer [73, 94].

FOXJ2 overexpression is associated with poor prognosis, progression, and metastasis in nasopharyngeal carcinoma [95]. FOXQ1, a member of the FOX family, is overexpressed in colorectal cancer, and it enhances tumorigenicity and tumor growth [96]. However, it has been reported that FOXJ2 suppresses migration and invasion in extrahepatic cholangiocarcinoma [97], hepatocellular carcinoma [98], glioma [99], and breast cancer [100]. Thus, FOXJ2 can promote or suppress malignancy depending on cancer type, which may account for the colorectal carcinoma-selective association of FOXJ2-Abs (Supplementary Table S1).

CPSF2 has a suppressive role in cell invasion in thyroid cancer and cancer stem cell population [101]. It is involved in the 6-gene prognostic signature for hepatocellular carcinoma overall survival prediction [102]. Our results showed only a slight association of CPSF2-Abs with esophageal squamous cell carcinoma (*P* < 0.01) but not with other types of cancer (Supplementary Table S1). CPSF2-Ab may reflect DM-caused atherosclerosis as described above, and the causes of cancer and atherosclerosis overlap with each other. Thus, CPSF2-Abs may be associated indirectly with some types of cancer.

### Characteristics of antibody biomarkers

Atherosclerosis progresses slowly over many years, finally leading to the onset of AIS or AMI. The prodromal stages of AIS and AMI may be accompanied by tissue destruction in arteries. The development of autoantibodies may be caused by high expressions of antigenic proteins in arteries followed by tissue destruction-induced exposure of antigens to immune cells. Repeated destruction/exposure can considerably increase antibody levels while keeping the antigen level low. Thus, antibody markers are much more sensitive than antigen markers. In addition, serum IgG proteins are highly stable and not easily degraded. As such, antibody markers are highly suitable for detecting trivial alterations caused by early-stage lesions. This is consistent with results that in this study, serum DIDO1-Ab, FOXJ2-Ab, and CPSF2-Ab were elevated not only in patients with AIS but also in those with TIA, a prodromal lesion of AIS (Fig. 2).

AIS is a severe disease that often leads to death. Once it occurs, even without death, affected patients require a long rehabilitation period, with this disease also being the first cause of being bedridden. However, if the onset of AIS is predicted, most patients can avoid it via an appropriate treatment. Therefore, the development of highly sensitive and predictive biomarkers is eagerly expected. We discovered that serum DIDO1-Ab, FOXJ2-Ab, and CPSF2-Ab are useful in predicting the onset of AIS (Table 8), although these three markers may not be sufficient to diagnose all AIS types. AIS is a multifactorial disease, and each marker may associated with a different cause. The more biomarkers are identified, the more precise predictions can be achieved. Further investigations may be necessary for practical use.

### Limitation

Although the present study suggests kidney failure-associated DIDO1-Ab, hypertension-related FOXJ2-Ab, and DM-related CPSF2-Ab markers as risk factors of AIS, further study using the increasing number of specimens is needed to verify the suggestion. Because our present study was carried out using specimens obtained from hospitals and universities in Japan, it is obscure whether our conclusion is generalized in other population. Further international collaborative research using the specimens from many countries is necessary for the practical use in the world.

## Conclusions

Serum DIDO1-Ab, FOXJ2-Ab, and CPSF2-Ab appear to be useful for diagnosing AIS and may originate from kidney disease, hypertension, and DM, respectively.

## Supplementary Information

**Additional file 1.** Supplementary information.

## Data Availability

The datasets used and analyzed during the current study are available from the corresponding author on reasonable request.

## Abbreviations

AIS: Acute ischemic stroke
AlphaLISA: Amplified luminescent proximity homogeneous assay-linked immunosorbent assay
AMI: Acute myocardial infarction
asympt-CI: Asymptomatic cerebral infarction
AUC: Area under the curve
bCPSF2-607: Biotinylated peptide of CPSF2 amino acids 607-621, biotin-QVRLKDSLVSSLQFC
bDIDO1-297: Biotinylated peptide of DIDO1 amino acids 297-314, biotin-AMAASKKTAPPGSAVGKQ
bFOXJ2-426: Biotinylated peptide of FOXJ2 amino acids 426-440, biotin-KMVNRLNWSSIEQSQ
BMP: Bone morphogenetic protein
cCI: Chronic-phase cerebral infarction
CI: Confidence interval
CKD: Chronic kidney disease
COPE: Coatomer protein complex subunit epsilon
CPSF2: Cleavage and polyadenylation specificity factor
CPSF2-Ab: Anti-bCPSF2-607 peptide antibody
CTEPH: Chronic thromboembolic pulmonary hypertension
CVD: Cardiovascular disease
DIDO1: Death-inducer obliterator 1
DIDO1-Ab: Anti-DIDO1 N-terminal protein antibody
DIDO1pep-Ab: Anti-bDIDO1-297 peptide antibody
DM: Diabetes mellitus
DSWMH: Deep and subcortical white matter hyperintensity
*E. coli*: *Escherichia coli*
FOXJ2: Forkhead box J2
FOXJ2-Ab: Anti-bFOXJ2-426 peptide antibody
GST: Glutathione S-transferase
HbA1c: Glycated hemoglobin
HD: Healthy donor
JPHC: Japan Public Health Center
max IMT: Maximum intima media thickness
OSA: Obstructive sleep apnea
PAH: Pulmonary arterial hypertension
PBS: Phosphate-buffered saline
ROC: Receiver operating characteristic
SD: Standard deviation
SEREX: Serological identification of antigens by cDNA expression cloning
SLE: Systemic lupus erythematosus
TGF-β: Transforming growth factor-β
TIA: Transient ischemic attack
