# Supplementary material for: Serum anti-DIDO1, anti-CPSF2, and anti-FOXJ2 antibodies as predictive risk markers for acute ischemic stroke
Source: BMC Med. 2021 Jun 9;19:131. doi: 10.1186/s12916-021-02001-9 (PMC8188684; doi:10.1186/s12916-021-02001-9)

**Additional file 1: Supplementary information**

**Supplementary Table S1** Comparison of serum DIDO1-Ab, FOXJ3-Ab, and CPSF2-Ab levels between healthy donors (HDs) and patients with cancer

The types of cancer diagnoses included colorectal carcinoma (CRC), esophageal squamous cell carcinoma (ESCC), gastric cancer (GC), breast cancer (Br-Ca), and pancreatic cancer (Panc-Ca). Purified GST-DIDO1:1-275 protein and peptides, bFOXJ2-426 and bCPSF2-607, were used as antigens for the evaluation of the antibody levels using AlphaLISA. The cutoff values were determined as the average HD values plus two standard deviations (SDs), and positive samples for which the alpha counts exceeded the cutoff value were scored. *P*-values were calculated using the Kruskal–Wallis test. *P*-values lower than 0.05 and positive rates higher than 10% are marked in bold. ns, not significant; –, the average antibody levels of patients were lower than those of control HDs.

| Alpha analysis (antibody level) | | DIDO1-Ab | FOXJ2-Ab | CPSF2-Ab |
| --- | --- | --- | --- | --- |
| HD | Average | 550 | 792 | 3,622 |
|  | SD | 647 | 759 | 2,628 |
|  | Cutoff value | 1,844 | 2,309 | 8,878 |
|  | Total No. | 125 | 125 | 125 |
|  | Positive No. | 5 | 6 | 6 |
|  | Positive (%) | 4.0% | 4.8% | 4.8% |
| CRC | Average | 467 | 1,422 | 4,090 |
|  | SD | 1,009 | 1,922 | 3,307 |
|  | Total No. | 249 | 250 | 252 |
|  | Positive No. | 9 | 48 | 21 |
|  | Positive (%) | 3.6% | **19.2%** | 8.3% |
|  | *P* (CRC vs HD) | – | **< 0.01** | ns |
| ESCC | Average | 379 | 874 | 4,876 |
|  | SD | 980 | 2,141 | 5,054 |
|  | Total No. | 91 | 92 | 93 |
|  | Positive No. | 4 | 10 | 14 |
|  | Positive (%) | 4.4% | **10.9%** | **15.1%** |
|  | *P* (ESCC vs HD) | – | ns | ns |
| GC | Average | 310 | 707 | 3,811 |
|  | SD | 945 | 1,756 | 3,813 |
|  | Total No. | 95 | 95 | 95 |
|  | Positive No. | 4 | 4 | 8 |
|  | Positive (%) | 4.2% | 4.2% | 8.4% |
|  | *P* (GC vs HD) | – | – | ns |
| Br-Ca | Average | 73 | 493 | 2,741 |
|  | SD | 638 | 1,141 | 2,094 |
|  | Total No. | 93 | 93 | 94 |
|  | Positive No. | 2 | 2 | 1 |
|  | Positive (%) | 2.2% | 2.2% | 1.1% |
|  | *P* (Br-Ca vs HD) | – | – | – |
| Panc-Ca | Average | 128 | 928 | 4,084 |
|  | SD | 1,082 | 1,618 | 5,005 |
|  | Total No. | 95 | 95 | 95 |
|  | Positive No. | 2 | 11 | 9 |
|  | Positive (%) | 2.1% | **11.6%** | 9.5% |
|  | *P* (Panc-Ca vs HD) | – | ns | ns |

**Supplementary Table S2** Comparison of serum antibody levels of HDs versus those of patients with autoimmune diseases

The types of autoimmune diseases examined were Sjögren's syndrome (SS), rheumatoid arthritis (RA), systemic lupus erythematosus (SLE), and ulcerative colitis (UC). Purified GST-DIDO1:1-275 protein and peptides, bFOXJ2-426 and bCPSF2-607, were used as antigens for evaluation of the antibody levels using AlphaLISA. The numbers indicated are as described in the legends of supplementary Table S1. ns, not significant; –, the average antibody levels of patients were lower than those of control HDs.

| Alpha analysis (antibody level) | | DIDO1-Ab | FOXJ2-Ab | CPSF2-Ab |
| --- | --- | --- | --- | --- |
| HD | Average | 890 | 1,916 | 2,899 |
|  | SD | 925 | 1,279 | 1,934 |
|  | Cutoff value | 2,739 | 4,474 | 6,768 |
|  | Total No. | 96 | 95 | 94 |
|  | Positive No. | 4 | 6 | 6 |
|  | Positive (%) | 4.2% | 6.3% | 6.4% |
| SS | Average | 938 | 2,650 | 1,554 |
|  | SD | 1,260 | 2,597 | 1,172 |
|  | Total No. | 55 | 55 | 54 |
|  | Positive No. | 3 | 7 | 0 |
|  | Positive (%) | 5.5% | **12.7%** | 0.0% |
|  | *P* (SS vs HD) | ns | ns | – |
| RA | Average | 1,358 | 2,702 | 3,966 |
|  | SD | 903 | 1,608 | 2,800 |
|  | Total No. | 94 | 93 | 93 |
|  | Positive No. | 6 | 13 | 13 |
|  | Positive (%) | 6.4% | **14.0%** | **14.0%** |
|  | *P* (RA vs HD) | **< 0.001** | **< 0.01** | **< 0.05** |
| SLE | Average | 2,065 | 3,848 | 2,811 |
|  | SD | 3,450 | 2,786 | 1,788 |
|  | Total No. | 95 | 93 | 94 |
|  | Positive No. | 15 | 23 | 4 |
|  | Positive (%) | **15.8%** | **24.7%** | 4.3% |
|  | *P* (SLE vs HD) | **< 0.001** | **< 0.001** | ns |
| UC | Average | 1,511 | 3,295 | 2,768 |
|  | SD | 1,664 | 5,148 | 4,114 |
|  | Total No. | 41 | 40 | 40 |
|  | Positive No. | 5 | 6 | 3 |
|  | Positive (%) | 12.2% | 15.0% | 7.5% |
|  | *P* (UC vs HD) | ns | ns | ns |

**Supplementary Table S3** Comparison of serum antibody levels of HDs versus those of patients with pulmonary diseases

The types of pulmonary diseases examined were chronic thromboembolic pulmonary hypertension (CTEPH), pulmonary arterial hypertension (PAH), and obstructive sleep apnea (OSA). Purified GST-DIDO1:1-275 protein and peptides, bFOXJ2-426 and bCPSF2-607, were used as antigens for evaluation of the antibody levels using AlphaLISA. The numbers indicated are as described in the legends of supplementary Table S1. ns, not significant; –, the average antibody levels of patients were lower than those of control HDs.

| Alpha analysis (antibody level) | | DIDO1-Ab | FOXJ2-Ab | CPSF2-Ab |
| --- | --- | --- | --- | --- |
| HD | Average | 6,174 | 6,255 | 6,380 |
|  | SD | 2,396 | 2,973 | 4,887 |
|  | Cutoff value | 10,965 | 12,200 | 16,153 |
|  | Total No. | 96 | 96 | 96 |
|  | Positive No. | 3 | 6 | 4 |
|  | Positive (%) | 3.1% | 6.3% | 4.2% |
| CTEPH | Average | 5,356 | 9,728 | 6,157 |
|  | SD | 2,618 | 5,230 | 6,098 |
|  | Total No. | 96 | 96 | 96 |
|  | Positive No. | 4 | 25 | 2 |
|  | Positive (%) | 4.2% | **26.0%** | 2.1% |
|  | *P* (CTEPH vs HD) | – | **< 0.001** | – |
| PAH | Average | 7,331 | 12,796 | 6,356 |
|  | SD | 3,301 | 8,153 | 5,773 |
|  | Total No. | 86 | 86 | 86 |
|  | Positive No. | 12 | 36 | 5 |
|  | Positive (%) | **14.0%** | **41.9%** | 5.8% |
|  | *P* (PAH vs HD) | ns | **< 0.001** | – |
| OSA | Average | 5,588 | 8,017 | 6,323 |
|  | SD | 2,771 | 5,628 | 5,368 |
|  | Total No. | 96 | 95 | 95 |
|  | Positive No. | 5 | 14 | 5 |
|  | Positive (%) | 5.2% | **14.7%** | 5.3% |
|  | *P* (OSA vs HD) | – | ns | – |

**Supplementary Table S4** Subjects’ information in the Sawara Hospital cohort used for correlation analysis

| Subjects' parameter* | Average ± SD** |
| --- | --- |
| Age | 68.5 ± 14.6 |
| Height | 158.0 ± 9.8 (cm) |
| Weight | 57.8 ± 12.1 (kg) |
| BMI | 23.0 ± 3.6 |
| max IMT | 2.32 ± 1.37 (mm) |
| A/G | 1.48 ± 0.30 |
| AST(GOT) | 24.7 ± 18.3 (U/L) |
| ALT(GPT) | 20.8 ± 14.2 (U/L) |
| ALP | 242 ± 85 (U/L) |
| LDH | 200 ± 106 (U/L) |
| tBil | 0.81 ± 0.36 (mg/dL) |
| CHE | 319.9 ± 79.8 (U/L) |
| γ-GTP | 33.3 ± 39.1 (U/L) |
| TP | 7.18 ± 0.55 (g/dL) |
| Albumin | 4.24 ± 0.51 (g/dL) |
| BUN | 16.3 ± 11.8 (mg/dL) |
| CRE | 0.82 ± 0.46 (mg/dL) |
| eGFR | 72.0 ± 21.1 (mL/min) |
| UA | 5.63 ± 8.45 (mg/dL) |
| AMY | 182 ± 108 (U/L) |
| T-CHO | 197 ± 36 (mg/dL) |
| HDL-C | 55.5 ± 15.5 (mg/dL) |
| TG | 119.9 ± 79.0 (mg/dL) |
| Na | 140.3 ± 2.8 (mmol/L) |
| K | 4.15 ± 0.42 (mol/L) |
| Cl | 104.6 ± 3.2 (mmol/L) |
| Ca | 9.22 ± 0.39 (mg/dL) |
| IP | 3.42 ± 0.49 (mg/dL) |
| Fe | 94.5 ± 35.3 (μg/dL) |
| CRP | 0.80 ± 2.63 (mg/dL) |
| LDL-C | 116.4 ± 31.0 (mg/dL) |
| WBC | 6.76 ± 2.53 (x 10^3^/mm) |
| RBC | 4.49 ± 0.57 (x 10^6^/mm) |
| HGB | 13.8 ± 1.8 (g/dL) |
| HCT | 40.5 ± 4.8 (%) |
| MCV | 90.4 ± 5.1 (fL) |
| MCH | 30.7 ± 2.1 (pg) |
| MCHC | 33.9 ± 1.2 (g/dL) |
| RDW | 13.3 ± 1.2 |
| PLT | 2.21 ± 0.73 (x 10^5^/mm) |
| MPV | 9.91 ± 0.77 (fL) |
| PCT | 0.22 ± 0.07 (ng/mL) |
| PDW | 11.2 ± 1.6 (fL) |
| BS | 125.8 ± 49.9 (mg/dL) |
| HbA1c | 5.76 ± 2.02 (%) |

*Subjects' parameters used were age, height, weight, body mass index (BMI), maximum intima–media thickness (max IMT), albumin/globulin ratio (A/G), aspartate aminotransferase (AST), alanine amino transferase (ALT), alkaline phosphatase (ALP), lactate dehydrogenase (LDH), total bilirubin (tBil), cholinesterase (CHE), γ-glutamyl transpeptidase (γ-GTP), total protein (TP), albumin, blood urea nitrogen (BUN), creatinine (CRE), estimated glomerular filtration rate (eGFR), uric acid (UA), amylase (AMY), total cholesterol (T-CHO), high-density lipoprotein cholesterol (HDL-C), triglyceride (TG), sodium (Na), potassium (K), chlorine (Cl), calcium (Ca), inorganic phosphate (IP), iron (Fe), C-reactive protein (CRP), low-density lipoprotein cholesterol (LDL-C), white blood cells (WBC), red blood cells (RBC), hemoglobin (HGB), hematocrit (HCT), mean corpuscular volume (MCV), mean corpuscular hemoglobin (MCH), MCH concentration (MCHC), red cell distribution width (RDW), platelets (PLT), mean platelet volume (MPV), procalcitonin (PCT), platelet distribution width (PDW), blood sugar (BS), and glycated hemoglobin (HbA1c).

**Average ± standard deviation (SD) for each parameter are shown.

**Supplementary Fig. S1** Comparison of serum anti-DIDO1-peptide antibody levels between HDs and patients with TIA or AIS

(a) Biotinylated DIDO1 peptide (bDIDO1-297) was used as the antigen. Serum antibody levels were determined by AlphaLISA and are shown as box-whisker plots, as described in the legend of Fig. 2. The total numbers and average values ± SDs of antibody levels were 285 and 4736 ± 3179 for HDs, 92 and 6950 ± 5251 for TIA, and 464 and 7309 ± 5415 for AIS. Responses to antibodies against DIDO1 peptide (b, c) were also evaluated using ROC analysis, and summarized in Table 5.


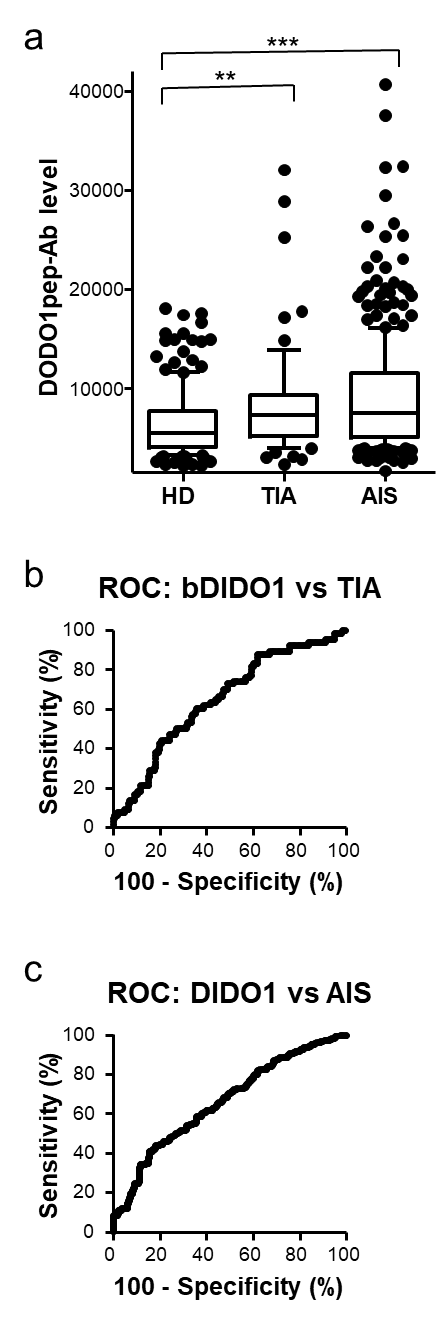

Supplement: Supplementary file 1 — Additional file 1. Supplementary information. [file 12916_2021_2001_MOESM1_ESM.docx]
